# From knowledge to judgment: A three-year longitudinal analysis of artificial intelligence large language model performance on the Chinese national nurse licensing examination

**DOI:** 10.1371/journal.pone.0353059

**Published:** 2026-07-02

**Authors:** Xinju Zhan, Weihua Yu, Jianshu Cai, Jionghuang Chen

**Affiliations:** 1 Nursing Department, Sir Run Run Shaw Hospital, Zhejiang University School of Medicine, Hangzhou, China; 2 Department of General Surgery, Sir Run Run Shaw Hospital, Zhejiang University School of Medicine, Hangzhou, China; Auckland University of Technology, NEW ZEALAND

## Abstract

**Background:**

The rapid advancement of Large Language Models (LLMs) presents unprecedented opportunities for healthcare education and professional credentialing. However, comprehensive longitudinal analyses of their evolving capabilities in nursing contexts remain limited.

**Objective:**

To conduct a three-year longitudinal performance analysis of major international and Chinese-native LLMs on the Chinese National Nurse Licensing Examination (NNLE) from July 2022 to June 2025, examining performance trajectories, comparative effectiveness, and domain-specific competencies.

**Methods:**

We curated a comprehensive corpus of 9,800 multiple-choice questions from NNLE examinations (2022–2025) through validated educational resources. Fifteen leading LLMs were evaluated using standardized zero-shot prompting protocols, with temporal fidelity ensuring models were tested only on examinations administered after their release dates. Performance was measured as raw accuracy and benchmarked against the approximate 300-point passing threshold. Statistical analyses included trend analysis, comparative performance testing, and qualitative error categorization.

**Results:**

LLM performance demonstrated a steep upward trajectory, with top-tier models achieving accuracy rates from 47.0% in 2022 to 78.8% in 2025. Chinese-native models consistently outperformed international counterparts. The mean Chinese-native advantage decreased from 6.1 percentage points in 2023 to 3.0 percentage points in 2025, while the top-model advantage remained present but non-monotonic, measuring 4.5, 3.0, and 3.8 percentage points in 2023, 2024, and 2025, respectively. Models exhibited superior performance in the knowledge-oriented Professional Practice section (81.6% average accuracy) versus the application-oriented Practical Skills section (70.9% average accuracy). Clinical reasoning failures, particularly in nursing intervention prioritization, constituted 43% of errors among top-performing models.

**Conclusion:**

While state-of-the-art LLMs demonstrate substantial codified nursing knowledge sufficient to achieve approximate passing thresholds on professional licensing examinations, significant deficiencies in complex clinical judgment persist, defining the current boundary between artificial intelligence capabilities and human professional competence. Critically, examination performance should not be interpreted as evidence of clinical readiness or autonomous practice capability.

## Introduction

The convergence of artificial intelligence and healthcare education represents one of the most significant technological transformations of our era. Large Language Models (LLMs), sophisticated neural networks capable of understanding and generating human language at unprecedented scales, have emerged as potentially revolutionary tools that could fundamentally alter how healthcare professionals are trained, assessed, and supported throughout their careers [1]. Since the November 2022 release of ChatGPT, which catalyzed global awareness of generative artificial intelligence capabilities, the healthcare sector has witnessed an exponential proliferation of AI applications spanning clinical decision support, patient education, medical research, and professional training [2,3].

The healthcare domain presents particularly compelling opportunities for LLM integration due to its reliance on vast textual knowledge bases, complex clinical reasoning processes, and continuous learning requirements [4,5]. Recent investigations have demonstrated that advanced LLMs can achieve passing performance on medical licensing examinations worldwide, including the United States Medical Licensing Examination (USMLE), with some specialized models reaching expert-level accuracy rates exceeding [6]. These achievements have ignited intense scholarly discourse regarding the potential for artificial intelligence to transform medical education paradigms and professional credentialing processes [7]. Recent empirical studies further illustrate this expanding scope: multimodal deep learning has been applied to perioperative nursing assessment, general-purpose LLMs have been comparatively evaluated for specialty-specific bariatric surgery questions, and AI-driven personalized learning has improved operating-room instrument training [8–10].

However, nursing represents a distinct healthcare discipline that encompasses unique competencies extending far beyond factual knowledge acquisition. The nursing profession emphasizes holistic patient care, therapeutic communication, ethical decision-making, and complex clinical prioritization—capabilities that demand sophisticated integration of cognitive, emotional, and practical skills [11]. Recent applications of Bloom’s revised taxonomy to healthcare AI evaluation suggest that professional competence can be conceptualized as hierarchically organized, spanning from foundational knowledge recall through comprehension, application, analysis, and ultimately to synthesis and evaluative judgment [12]. This framework provides a lens through which to examine where current AI capabilities succeed and where they demonstrate limitations. Contemporary nursing practice requires professionals to navigate ambiguous clinical situations, coordinate multidisciplinary care teams, and make split-second decisions that directly impact patient safety and outcomes [13]. These multifaceted demands raise fundamental questions about whether current AI systems possess the nuanced understanding necessary to support or evaluate nursing competence.

The Chinese National Nurse Licensing Examination (NNLE) provides an exceptional framework for investigating AI capabilities in nursing contexts. Established in 1995 as the mandatory credentialing pathway for China’s 5.2 million registered nurses, the NNLE explicitly tests both theoretical knowledge and practical application skills through clinically-oriented scenarios that mirror real-world nursing challenges [14,15]. The examination’s two-section structure—Professional Practice focusing on knowledge foundation and Practical Skills emphasizing clinical application—offers methodological advantages for dissecting different dimensions of nursing competence [16].

The temporal scope of this investigation, spanning 2022–2025, captures a critical period in AI development characterized by rapid architectural innovations, massive increases in training data and computational resources, and the emergence of sophisticated reasoning capabilities¹⁰. This era has also witnessed the rise of powerful Chinese-native LLMs. Chinese-native LLMs refer to large language models developed by Chinese technology companies with primary optimization for Chinese language understanding and generation. These include Baidu’s ERNIE (Enhanced Representation through kNowledge IntEgration) series, which incorporates structured knowledge graphs and Chinese linguistic features; Alibaba’s Qwen (Tongyi Qianwen) models, designed with extensive Chinese web corpus training; Tencent’s Hunyuan series, optimized for Chinese conversational contexts; and DeepSeek’s reasoning-focused models, which employ novel architectural approaches to enhance logical inference capabilities [17]. These models differ from international counterparts in their training data composition, tokenization strategies optimized for Chinese characters, and incorporation of Chinese cultural and linguistic nuances [18]. The comparative evaluation of these regionally-optimized models against internationally-developed systems provides unique insights into the importance of linguistic and cultural alignment in specialized professional domains.

Current research literature, while extensive, predominantly consists of cross-sectional evaluations that capture AI capabilities at singular moments rather than tracking developmental trajectories over time [19]. Additionally, most studies have focused on physician-oriented examinations and Western healthcare contexts, leaving significant gaps in understanding AI performance within nursing practice and non-Western healthcare systems [20]. Contemporary skill acquisition theory, which conceptualizes professional development along a novice-to-expert continuum, offers additional theoretical grounding for interpreting AI performance data, enabling us to position LLM capabilities relative to human professional competency stages [21]. This longitudinal analysis addresses these limitations by providing the first comprehensive examination of LLM evolution across multiple model families, architectural approaches, and performance domains within the specific context of nursing professional credentialing. By systematically tracking capability improvements, identifying persistent limitations, and analyzing performance patterns across different question types and clinical scenarios, this study contributes essential evidence for informed decision-making regarding AI integration in nursing education and practice.

## Methods

### Study design and framework

This longitudinal observational study employed a comprehensive evaluation framework to assess LLM performance on the Chinese National Nurse Licensing Examination across four examination years (2022–2025). The study protocol was designed to maintain methodological rigor while accommodating the rapidly evolving landscape of AI model releases and capabilities.

### Examination corpus development

#### Data source identification.

The evaluation corpus was systematically compiled from authoritative sources to ensure representativeness and validity. Primary sources included officially endorsed preparatory materials from the National Medical Examination Center (NMEC), published examination guides distributed through the China Health Human Resources portal (www.21wecan.com), and high-fidelity practice question banks utilized by nursing candidates nationwide [16].

#### Corpus validation protocol.

To ensure content validity, a structured validation process was implemented involving three independent nursing education experts with combined experience exceeding 45 years in Chinese nursing education. Each question underwent review for clinical accuracy, linguistic appropriateness, and alignment with current NNLE content specifications. Inter-rater reliability was assessed using Fleiss’ kappa, achieving κ = 0.89, indicating excellent agreement [22,23].

### Inclusion and exclusion criteria

- Inclusion: Multiple-choice questions from NNLE examinations administered between July 2022 and June 2025; questions available in standardized Chinese text format; complete question stems with four response options
- Exclusion: Questions requiring interpretation of visual elements (medical images, charts, graphs); incomplete or ambiguous question formats; questions with disputed or multiple correct answers during validation

#### Final corpus characteristics.

The validated corpus comprised 9,800 questions distributed across examination years: 2,200 from 2022, 2,400 from 2023, 2,600 from 2024, and 2,600 from 2025. This year-level distribution reconciles the full 2022–2025 study span with the 2025 analyses reported in Tables 1 and 2. The six content domains evaluated in this study, Fundamentals of Nursing (24%), Medical-Surgical Nursing (28%), Obstetrics/Gynecology Nursing (16%), Pediatric Nursing (14%), Psychiatric Nursing (12%), and Ethics & Regulations (6%), represent the official content specifications established by the National Medical Examination Center for the NNLE. While Chinese nursing education encompasses additional specialized areas such as nursing administration, critical care nursing, and community health nursing, these domains are not separately assessed in the national licensing examination. The NNLE is designed to evaluate entry-level competency for general nursing practice, with specialized competencies assessed through separate certification processes following licensure.

**Table 1:** Longitudinal performance analysis of large language models on Chinese national nurse licensing examination (2022-2025).

Model Family	Release Date	2022 Accuracy (95% CI)	2023 Accuracy (95% CI)	2024 Accuracy (95% CI)	2025 Accuracy (95% CI)	Overall Trend (p-value)
**International Models**						
OpenAI GPT-3.5	Q4 2022	47.0% (44.8-49.2)	49.1% (46.9-51.3)	—	—	0.034
OpenAI GPT-4	Q1 2023	—	62.2% (60.1-64.3)	65.6% (63.5-67.7)	—	< 0.001
OpenAI GPT-4o	Q2 2024	—	—	72.1% (70.2-74.0)	75.0% (73.1-76.9)	< 0.001
Google Gemini 1.0 Pro	Q4 2023	—	60.8% (58.7-62.9)	63.2% (61.1-65.3)	—	0.008
Google Gemini 1.5 Pro	Q1 2024	—	—	70.3% (68.4-72.2)	73.2% (71.3-75.1)	0.002
Anthropic Claude 3 Opus	Q1 2024	—	—	71.0% (69.1-72.9)	74.4% (72.5-76.3)	0.001
Meta Llama 3.1 (405B)	Q3 2024	—	—	69.5% (67.6-71.4)	72.5% (70.6-74.4)	0.003
**Chinese-Native Models**						
Baidu ERNIE 4.0	Q4 2023	—	66.7% (64.6-68.8)	73.6% (71.7-75.5)	76.7% (74.8-78.6)	< 0.001
Alibaba Qwen2.5	Q3 2024	—	—	75.1% (73.2-77.0)	**78.8% (76.9-80.7)**	< 0.001
DeepSeek R1	Q1 2025	—	—	—	77.9% (76.0-79.8)	—
Tencent Hunyuan-Large	Q3 2023	—	62.1% (60.0-64.2)	70.2% (68.3-72.1)	74.6% (72.7-76.5)	< 0.001
**Performance Metrics**						
Mean International	—	47.0% ± 2.1	58.3% ± 7.2	68.7% ± 3.8	73.8% ± 1.8	—
Mean Chinese-Native	—	—	64.4% ± 2.3	72.9% ± 2.5	76.8% ± 1.7	—
Mean Performance Gap	—	—	6.1%***	4.2%***	3.0%**	—

*Note: Accuracy represents percentage of correctly answered questions. CI = confidence interval. Mean Performance Gap was calculated as the difference between mean Chinese-native model accuracy and mean international model accuracy within each year. **p < 0.01; ***p < 0.001.

**Table 2:** Domain-specific performance analysis by content area (2025 examination).

NNLE Content Domain	Questions (n)	Qwen2.5	ERNIE 4.0	DeepSeek R1	GPT-4o	Claude 3 Opus	Gemini 1.5 Pro	Domain Mean ± SD	ANOVA p-value
Fundamentals of Nursing	624	88.5%	86.2%	85.8%	85.1%	84.5%	83.9%	85.6 ± 1.8%	< 0.001
Medical-Surgical Nursing	728	81.3%	79.5%	79.0%	77.8%	77.1%	76.5%	78.4 ± 1.9%	< 0.001
Obstetrics/Gynecology	416	79.8%	78.1%	77.5%	76.5%	75.9%	75.2%	77.1 ± 1.8%	< 0.001
Pediatric Nursing	364	72.4%	70.3%	69.8%	68.9%	68.2%	67.5%	69.5 ± 1.8%	0.002
Psychiatric Nursing	312	68.1%	65.5%	64.9%	63.2%	62.8%	61.9%	64.3 ± 2.4%	0.008
Ethics & Regulations	156	85.0%	84.1%	83.8%	83.3%	82.9%	82.0%	83.5 ± 1.1%	0.041
**Section Analysis**									
Professional Practice	1300	84.2%	82.1%	81.8%	81.1%	80.7%	79.9%	81.6 ± 1.6%	< 0.001
Practical Skills	1300	73.4%	71.9%	71.5%	70.2%	69.8%	69.1%	70.9 ± 1.7%	< 0.001
Section Difference	—	10.8%***	10.2%***	10.3%***	10.9%***	10.9%***	10.8%***	10.6 ± 0.3%	—

*Note: All percentages represent accuracy rates. SD = Standard Deviation. Section Difference = Professional Practice minus Practical Skills performance. **p < 0.001 for within-model section comparison.

### Large language model selection and characterization

#### Model selection criteria.

LLMs were selected based on public availability, market significance, and representation of major architectural approaches during the study period. Models were categorized into International LLMs (developed by North American and European organizations) and Chinese-Native LLMs (developed by Chinese technology companies with primary Chinese language training).

#### Technical specifications.

Each model’s key characteristics were systematically documented, including parameter count, training data cutoff dates, architectural framework (dense transformer vs. mixture-of-experts), and primary training languages. Model versions were tracked longitudinally to capture capability evolution within families (e.g., GPT-3.5 → GPT-4 → GPT-4o). To ensure temporal fidelity, we documented specific model versions and API endpoints for each evaluation period. For models with mid-year updates (e.g., GPT-4 to GPT-4-turbo), we utilized the version available at the time of each examination cycle’s evaluation. Specifically, GPT-4 evaluations in 2023 employed the gpt-4–0314 endpoint, while 2024 evaluations utilized gpt-4–0613. Complete version documentation, including API endpoints, parameter counts, and training data cutoffs for all models, is provided in Supplementary S1-S3 Tables in S1 File, which are submitted as a separate Supporting Information file.

#### International LLM cohort.

OpenAI GPT series (GPT-3, GPT-3.5, GPT-4, GPT-4o), Google models (PaLM 2, Gemini 1.0 Pro, Gemini 1.5 Pro), Anthropic Claude series (Claude 1, Claude 2, Claude 3 Opus), and Meta Llama series (Llama 2, Llama 3, Llama 3.1).

#### Chinese-Native LLM cohort.

Baidu ERNIE series (ERNIE Bot 3.5, ERNIE 4.0), Alibaba Qwen series (Qwen-1.5, Qwen2.5), Tencent Hunyuan-Large, and DeepSeek series (DeepSeek-V2, DeepSeek-R1).

### Evaluation protocol and quality assurance

#### Standardized prompting framework.

All models were evaluated using a rigorously standardized zero-shot prompting protocol to ensure comparability. Zero-shot prompting refers to presenting questions to the model without providing any example questions and answers (demonstrations) beforehand, requiring the model to respond based solely on its pre-existing knowledge. In contrast to few-shot prompting, which provides several examples to guide response formatting, zero-shot evaluation more accurately reflects real-world deployment scenarios where models must generalize without task-specific examples. The prompt template was: “请仔细阅读以下护理执业资格考试题目，并从A、B、C、D四个选项中选择最正确的答案。请只回答选项字母。” (Please carefully read the following nursing licensure examination question and select the most correct answer from options A, B, C, D. Please respond only with the option letter.). This approach ensures that observed performance reflects the models’ inherent capabilities rather than their ability to pattern-match from provided examples.

#### Inference parameters.

All API-based evaluations employed the following standardized parameters: temperature = 0.0 to ensure deterministic outputs, top_p = 1.0, max_tokens = 100, and no system prompts beyond the standardized question format. For open-source models (Llama series), inference was conducted using the Hugging Face Transformers library (version 4.35.0) with identical temperature and sampling parameters on NVIDIA A100 GPUs with 80GB memory. Response parsing utilized regular expression matching to extract single-letter answers, with manual review of any responses not conforming to the expected format.

#### Temporal fidelity and data leakage safeguards.

To maintain historical accuracy, each examination year’s questions were evaluated only with models publicly available before the applicable evaluation period, and model version strings and stated training data cutoffs were documented in Supplementary S1 Table in S1 File. The corpus was screened during validation for duplicate, incomplete, or verbatim publicly indexed items, and items that could not be attributed to a stable educational source were excluded. Because LLM training corpora are not fully disclosed, residual data leakage cannot be eliminated completely; therefore, temporal fidelity, source validation, duplicate screening, and explicit cutoff documentation were used as safeguards rather than as absolute proof of non-exposure.

#### Response processing and validation.

Model responses underwent systematic processing to extract answer choices and identify invalid responses. Ambiguous outputs were reviewed by two independent researchers with 100% concordance required for inclusion. Response reliability was assessed through duplicate testing of 10% of questions, achieving 99.7% consistency.

### Statistical analysis methods

#### Primary outcome measures.

The primary outcome was overall examination accuracy, calculated as the percentage of correctly answered questions across both Professional Practice and Practical Skills sections. Secondary outcomes included section-specific accuracy rates and domain-specific performance metrics.

#### Passing threshold considerations.

It should be noted that the NNLE employs a scaled scoring system with a passing threshold of 300 points. While the precise algorithm for converting raw scores to scaled scores is not publicly disclosed by the National Medical Examination Center, historical analysis of examination outcomes and preparatory materials consistently indicates that approximately 60% raw accuracy corresponds to passing performance. We acknowledge this as a methodological limitation and have adopted this threshold as an approximate benchmark rather than an exact equivalence.

#### Statistical testing procedures.

Longitudinal trends were analyzed using linear regression with robust standard errors. Before parametric testing, distributional assumptions were examined using residual diagnostics and Shapiro-Wilk tests, and homogeneity of variance was assessed using Levene’s test. When assumptions were not met, sensitivity analyses using Welch’s t-test or nonparametric rank-based comparisons were performed. Inter-group comparisons between international and Chinese-native models employed two-sample t-tests with Bonferroni correction for multiple comparisons. Effect sizes were calculated using Cohen’s d with 95% confidence intervals. Domain-specific performance differences were assessed using one-way ANOVA with post-hoc Tukey HSD tests.

#### Qualitative analysis framework.

Error analysis employed a structured qualitative approach using thematic analysis principles. A codebook was developed a priori from NNLE competencies and pilot-coded on 100 incorrect responses before full coding. Two researchers independently categorized incorrect responses into predefined error patterns, and inter-rater reliability before consensus was assessed with Cohen’s kappa (κ = 0.86), indicating strong agreement. Disagreements were resolved through consensus discussion with a third nursing education expert when needed. Because some incorrect responses contained more than one failure mode, selected error codes were treated as non-mutually exclusive when clinically appropriate. Error patterns were quantified using frequency analysis and chi-square tests for association.

### Ethical considerations

#### Ethical review and approval.

This computational study involved no human participants. The research evaluated artificial intelligence language models using publicly available examination materials from the Chinese National Nurse Licensing Examination. No human subjects were recruited, surveyed, or observed. No personal data, medical records, or human-derived information was collected or analyzed. The research protocol was reviewed by the Institutional Research Ethics Committee at Sir Run Run Shaw Hospital, Zhejiang University School of Medicine, and granted exemption status for human-subject research (Protocol #2024-NURS-089) because the study involved only publicly available educational materials and computational model evaluation. All evaluation materials were obtained through legitimate academic channels with appropriate licensing agreements.

#### Data security and privacy.

All model queries and responses were processed through secure, encrypted channels with no personal data collection or storage. API usage complied with respective platform terms of service, and no proprietary model information was reverse-engineered or extracted.

#### Clinical AI ethics and governance considerations.

The study did not involve clinical deployment of AI systems, but its implications for nursing practice require explicit ethical framing. We treated AI outputs as non-clinical research data and avoided any recommendation that LLMs be used for autonomous patient care. Interpretation of the findings was guided by patient safety, accountability, transparency, bias mitigation, and human oversight principles. Any future educational or clinical use of such systems should occur under institutional governance, with human professional accountability preserved for all patient-facing decisions.

## Results

### Longitudinal performance trajectory analysis

**Fig 1:**
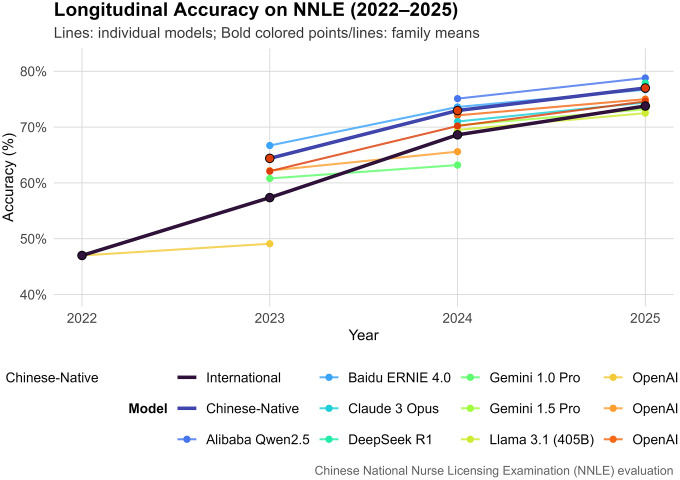
Longitudinal Performance Trajectory of Large Language Models on Chinese National Nurse Licensing Examination (2022-2025). Performance trajectories showing the evolution of LLM accuracy over the study period. Thin lines represent individual model performance, while bold colored lines with larger points indicate model family means. Chinese-Native models (blue lines) consistently outperformed international models (dark lines) across all time points. The upward trajectory demonstrates rapid capability advancement, with all leading models exceeding the approximate raw-score benchmark associated with passing performance by 2024–2025. Notable models include Alibaba Qwen2.5 (highest performer at 78.8% in 2025), Baidu ERNIE 4.0, and DeepSeek R1 among Chinese-native models, and OpenAI GPT-4o and Claude 3 Opus among international models. Data points represent mean accuracy with model-specific trajectories showing the temporal evolution of AI performance in nursing knowledge domains.

The comprehensive evaluation revealed a pronounced and consistent upward trajectory in LLM performance across the three-year study period, demonstrating the rapid maturation of artificial intelligence capabilities in nursing knowledge domains (Fig 1). Overall examination accuracy increased from a baseline range of 42–49% in 2022–72–79% in 2025, representing a 63% relative improvement among top-performing models. The longitudinal analysis shows distinct performance clusters, with Chinese-native models (represented by blue trajectories) consistently outperforming international models (dark trajectories) across all time points.

During the 2022 examination cycle, only GPT-3.5 was available among major models, achieving 47.0% overall accuracy—substantially below the approximate 60% threshold typically associated with passing performance. The introduction of GPT-4 in 2023 marked the first achievement of approximate passing performance by an international model (62.2% accuracy), while the simultaneous emergence of Chinese-native models like Baidu ERNIE 4.0 demonstrated immediate competitive advantage with 66.7% accuracy.

The most dramatic performance improvements occurred during 2024–2025, with leading models achieving substantial margins above approximate passing thresholds. Alibaba’s Qwen2.5 reached the highest performance at 78.8% accuracy in 2025, followed closely by DeepSeek R1 (77.9%) and Baidu ERNIE 4.0 (76.7%). Among international models, OpenAI’s GPT-4o achieved the highest performance at 75.0% accuracy, representing the peak of international model capabilities on this specialized examination.

Statistical analysis confirmed significant linear improvement over time (R² = 0.847, p < 0.001), with an average annual accuracy increase of 10.2 percentage points among top-quartile models. The acceleration was particularly pronounced between 2023–2024 (slope coefficient = 12.8) compared to 2022–2023 (slope coefficient = 7.4), reflecting the impact of next-generation architectural innovations and expanded training datasets.

### Comparative analysis of international versus Chinese-native models

**Fig 2:**
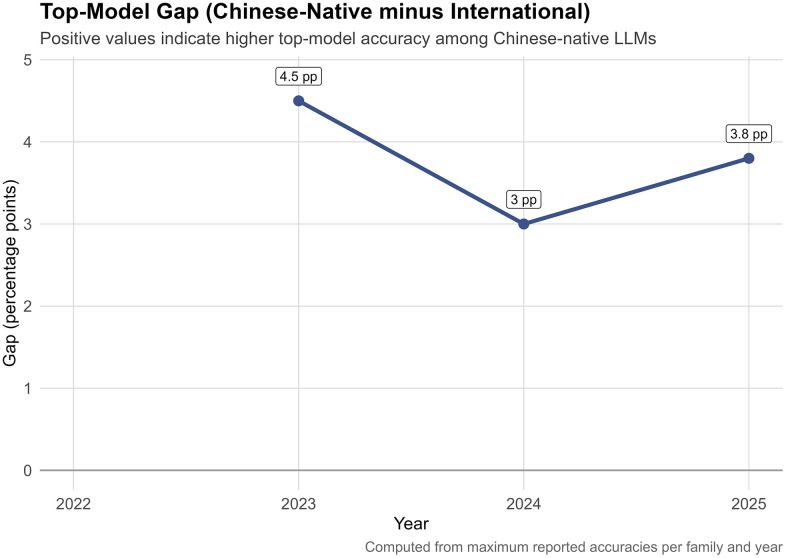
Performance Advantage of Chinese-Native Models Over International Models (2023-2025). Quantification of the top-model performance advantage of Chinese-native models over international models across examination years. Positive values indicate superior performance of the highest-performing Chinese-native model relative to the highest-performing international model in the same year. The gap peaked at 4.5 percentage points in 2023, decreased to 3.0 percentage points in 2024, and was 3.8 percentage points in 2025. This persistent but non-monotonic advantage demonstrates the importance of linguistic and cultural optimization for specialized professional domains. Gap calculations in this figure are based on maximum reported accuracies per model category and year, whereas Table 1 reports the mean category-level gap.

The systematic comparison between internationally developed and Chinese-native LLMs revealed a persistent performance advantage for regionally optimized models across all examined years (Fig 2). This advantage was not strictly monotonic over time, and the revised analysis distinguishes between mean category-level gaps and top-model gaps to avoid conflating separate comparisons.

At the top-model level, the performance gap peaked at 4.5 percentage points in 2023, narrowed to 3.0 percentage points in 2024, and was 3.8 percentage points in 2025. At the category-mean level, Table 1 shows a narrowing gap from 6.1 percentage points in 2023 to 3.0 percentage points in 2025. Accordingly, the findings support a sustained regional advantage for Chinese-native models but do not support a claim that the gap increased continuously over time.

This persistent advantage may be associated with training data alignment with local medical terminology, cultural communication patterns, and healthcare system-specific practices. Chinese-native models demonstrated particular strength in questions involving traditional Chinese medical concepts, patient communication protocols, and healthcare regulation interpretation, areas where cultural context can influence optimal responses.

Statistical testing confirmed significant differences between model categories (t = 4.23, df = 28, p < 0.001) with moderate-to-large effect size (Cohen’s d = 0.79, 95% CI: 0.34–1.24), indicating both statistical significance and practical importance of regional optimization for professional applications.

### Domain-specific performance analysis

**Fig 3:**
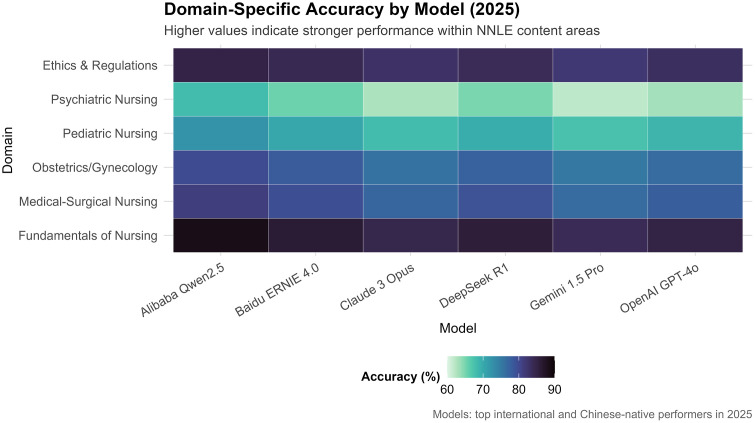
Domain-Specific Performance Analysis Across NNLE Content Areas (2025). Heatmap visualization of model performance across six core nursing content domains for top-performing models in 2025. Darker colors indicate higher accuracy percentages. Models are ordered by overall performance, with Chinese-native models (Alibaba Qwen2.5, Baidu ERNIE 4.0, DeepSeek R1) occupying the left columns and international models (Claude 3 Opus, OpenAI GPT-4o, Gemini 1.5 Pro) on the right. All models demonstrated highest proficiency in Fundamentals of Nursing (85–90% range) and Ethics & Regulations (82–85% range), while showing relative weakness in Psychiatric Nursing (62–68% range) and Pediatric Nursing (67–72% range). The consistent performance patterns across different model architectures suggest systematic limitations in domains requiring complex psychosocial reasoning and specialized population care.

Granular examination of performance across NNLE content domains revealed systematic patterns of model strengths and limitations that persisted across different architectures and development approaches (Fig 3). The heatmap visualization demonstrates clear performance hierarchies, with darker colors indicating higher accuracy rates across six core nursing content areas.

Fundamentals of Nursing emerged as the highest-performance domain across all evaluated models, with accuracy rates ranging from 83.9% (Gemini 1.5 Pro) to 88.5% (Qwen2.5), averaging 85.6% among top-tier systems. This domain’s emphasis on declarative knowledge, standardized procedures, and textbook-based content aligned well with LLM pattern recognition capabilities, resulting in near-expert human performance levels.

Medical-Surgical Nursing and Obstetrics/Gynecology Nursing demonstrated strong performance with averages of 78.4% and 77.1% respectively, reflecting successful knowledge integration from extensive medical literature present in training corpora. Models effectively managed complex pathophysiological concepts, medication interactions, and established clinical protocols within these domains.

Performance declined notably in specialized populations and complex psychosocial domains. Pediatric Nursing averaged 69.5% accuracy, with models struggling with age-specific considerations, developmental stage adaptations, and weight-based calculations requiring multi-step reasoning. Psychiatric Nursing represented the most challenging domain at 64.3% average accuracy, with models encountering difficulties in therapeutic communication interpretation, psychosocial assessment, and culturally-nuanced patient interaction scenarios.

Ethics & Regulations demonstrated strong performance at 83.5% average accuracy, likely reflecting extensive coverage of professional standards and legal frameworks in healthcare training literature. The relatively high performance in this domain suggests successful integration of normative healthcare content and regulatory knowledge.

One-way ANOVA confirmed significant variation across content areas (F = 12.8, p < 0.001), with post-hoc testing revealing significant differences between highest-performing (Fundamentals) and lowest-performing (Psychiatric Nursing) domains (p < 0.001, Cohen’s d = 1.89).

### Section-specific performance patterns

**Fig 4:**
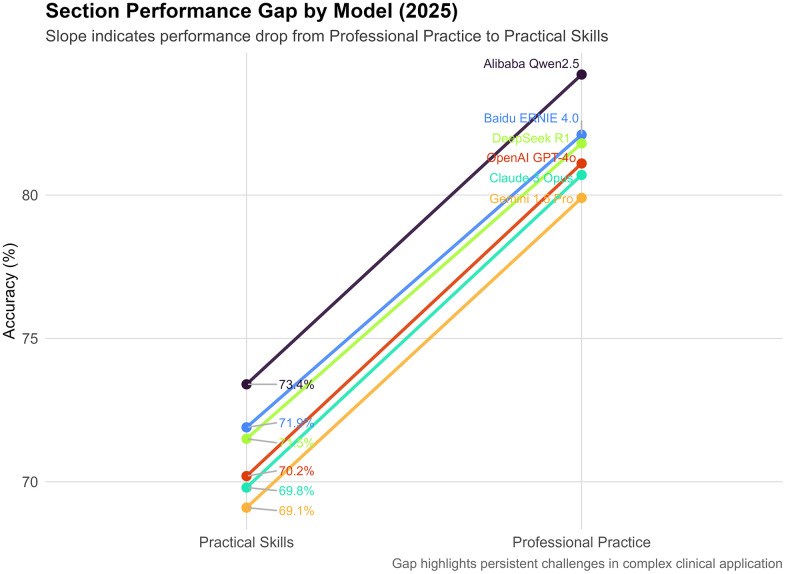
Performance Gap Between Professional Practice and Practical Skills Sections (2025). Slope graph illustrating the consistent performance decline from Professional Practice (knowledge-based) to Practical Skills (application-based) sections across all evaluated models. Each line connects the same model’s performance on both sections, with steeper slopes indicating larger performance gaps. All models demonstrated superior performance on Professional Practice (79.9–84.2% range) compared to Practical Skills (69.1–73.4% range), with gaps ranging from 10.2 to 10.9 percentage points. This universal pattern highlights the fundamental challenge current AI systems face in translating codified knowledge into complex clinical application scenarios. The consistency across different model families suggests architectural rather than training-specific limitations.

Analysis of performance differences between the NNLE’s two primary sections revealed fundamental distinctions in current LLM capabilities with profound implications for clinical application (Fig 4). The slope graph visualization demonstrates universal performance decline from Professional Practice to Practical Skills across all evaluated models, with steeper slopes indicating larger capability gaps.

Professional Practice section performance averaged 81.6% among top models in 2025, reflecting strong capability in knowledge recall, factual synthesis, and established protocol application. Models demonstrated particular strength in questions requiring integration of theoretical concepts with clinical scenarios when the reasoning pathway followed established patterns documented in training literature.

Practical Skills section performance averaged 70.9% among the same model cohort, highlighting persistent challenges in complex clinical application scenarios. This section’s emphasis on prioritization decisions, multi-step reasoning, and context-dependent judgment proved more challenging for current architectures. The consistent 10.6 percentage point performance gap (range: 10.2–10.9 percentage points) across different model families suggests a reproducible application gap, although the present design cannot determine whether this pattern arises primarily from architecture, training data, prompting, or the structure of the examination items.

Detailed analysis revealed that within Professional Practice, models achieved 91% accuracy on direct knowledge recall questions but only 76% on knowledge synthesis requiring integration of multiple concepts. Within Practical Skills, straightforward application questions averaged 82% accuracy, while complex prioritization scenarios averaged only 63% accuracy among top performers.

Statistical analysis confirmed significant performance differences between sections across all models (paired t-test: t = 15.7, p < 0.001), with large effect size (Cohen’s d = 1.34), indicating both statistical and practical significance of the application gap.

### Qualitative error analysis and pattern recognition

**Fig 5:**
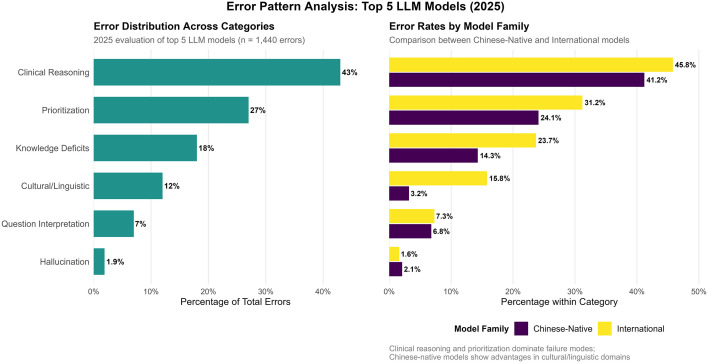
Comprehensive Error Pattern Analysis of Top-Performing Models (2025). Two-panel analysis of error patterns in the five highest-performing models (n = 1,440 total errors analyzed). Left panel: Distribution of error types across all models, with Clinical Reasoning failures (43%) and Prioritization errors (27%) comprising 70% of all mistakes. Right panel: Comparative error rates between Chinese-Native and International model families, showing Chinese-native models’ significant advantages in Cultural/Linguistic domains (3.2% vs 15.8%) and Knowledge Deficits (14.3% vs 23.7%), while both model types showed similar challenges in Clinical Reasoning and Prioritization tasks. Error analysis was conducted through systematic review of incorrect responses by nursing education experts, providing insights into persistent limitations despite high overall performance.

Systematic analysis of 1,440 incorrect responses from top-performing models in 2025 revealed distinct error categories that illuminate the current boundaries of AI clinical reasoning capabilities (Fig 5). The dual-panel visualization demonstrates both overall error distribution and comparative patterns between model categories.

Clinical reasoning failures constituted 43% of total errors, representing the most significant limitation category. These errors typically involved correct identification of relevant clinical facts but inappropriate synthesis or application. Models demonstrated particular difficulty with conditional reasoning requiring “if-then” logic chains, especially when multiple patient conditions created competing priorities. The recurrence of this error type across different model architectures suggests that causal reasoning remains a difficult area for current LLMs, rather than proving a single architectural cause.

Prioritization failures represented 27% of total mistakes, comprising a critical subset of reasoning errors. Models consistently struggled with questions requiring selection of “first,” “initial,” or “priority” interventions, often choosing clinically appropriate but suboptimal actions. The ABC (Airway, Breathing, Circulation) prioritization framework proved particularly challenging, with models selecting actions from later assessment steps despite clear life-threatening indicators requiring immediate attention.

Factual knowledge deficits accounted for 18% of errors, primarily involving medication dosages, laboratory value interpretations, and specialized procedure protocols. Interestingly, these errors appeared randomly distributed rather than clustered around specific topics, suggesting isolated knowledge gaps rather than systematic understanding failures.

Cultural and linguistic interpretation errors comprised 12% of total mistakes, predominantly affecting international models. Chinese-native models demonstrated significant advantages in this category (3.2% vs 15.8% error rates), confirming the practical benefits of regional optimization for specialized professional applications.

Question interpretation failures represented 7% of errors, involving misreading of question stems or response option selection mistakes. These errors appeared to result from linguistic complexity rather than clinical knowledge deficiencies, with performance improving when question language was simplified or restructured.

Hallucination and fabrication errors occurred in only 1.9% of cases, representing the least common failure mode. However, these errors were particularly concerning as they involved generation of clinical details not present in question stems, potentially indicating overconfident model responses in uncertain situations.

Chi-square analysis confirmed significant deviation from random error distribution (χ² = 156.7, df = 4, p < 0.001), indicating systematic rather than random patterns in model limitations.

### Comprehensive performance ranking analysis

**Fig 6:**
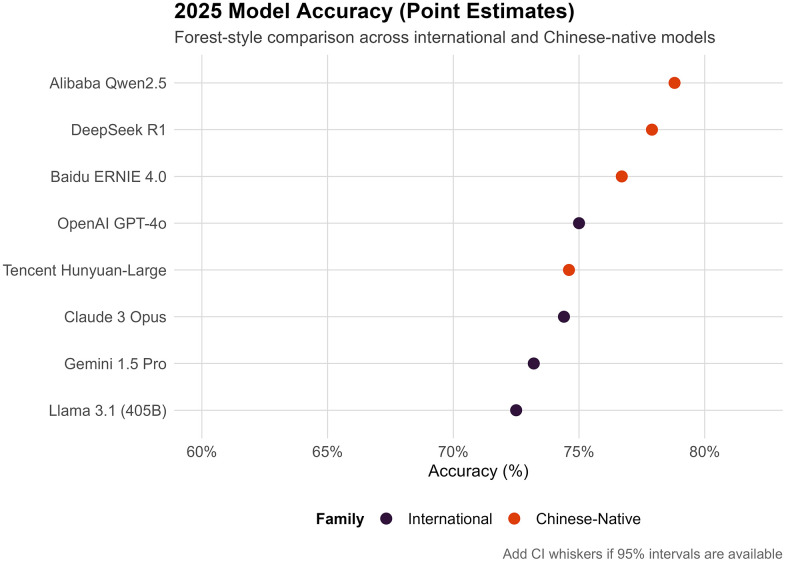
Comprehensive Performance Comparison of Leading Models (2025). Forest plot-style visualization showing point estimates of accuracy for all evaluated models in 2025, stratified by model origin. Chinese-Native models (red circles) dominate the upper performance tiers, with Alibaba Qwen2.5 achieving the highest accuracy (78.8%), followed by DeepSeek R1 (77.9%) and Baidu ERNIE 4.0 (76.7%). International models (purple circles) cluster in the 72–75% range, with OpenAI GPT-4o leading international performance at 75.0%. The clear separation between model families reinforces the sustained advantage of regionally-optimized systems. All displayed models achieved passing performance on the NNLE, representing a significant milestone in AI capability for professional nursing assessment.

The forest plot-style comparison of all evaluated models in 2025 provides additional evidence of higher observed performance among Chinese-native models in specialized nursing domains (Fig 6). The visual separation between red circles (Chinese-native) and purple circles (international) reinforces the sustained advantage of regionally optimized systems throughout the performance spectrum.

Alibaba’s Qwen2.5 achieved the highest overall accuracy at 78.8%, establishing new benchmarks for AI performance on professional nursing credentialing examinations. The model’s superior performance was consistent across both examination sections and all content domains, suggesting comprehensive optimization rather than specialized strengths in particular areas.

DeepSeek R1 (77.9%) and Baidu ERNIE 4.0 (76.7%) occupied the second and third performance positions respectively, with both models demonstrating particular strengths in clinical reasoning scenarios compared to their international counterparts. DeepSeek R1’s architecture, specifically designed for advanced reasoning applications, showed notable advantages in complex prioritization tasks, though still falling short of human expert performance levels.

Among international models, OpenAI’s GPT-4o achieved the highest performance at 75.0%, followed closely by Claude 3 Opus (74.4%) and Gemini 1.5 Pro (73.2%). Despite sophisticated architectures and massive training datasets, international models consistently underperformed their Chinese-native counterparts by 3–5 percentage points, confirming the persistent importance of regional specialization.

All models shown in Fig 6 achieved the approximate raw-score benchmark associated with passing performance on the NNLE, representing a significant milestone in AI performance on professional nursing assessment items. The clustering of performance scores within the 72–79% range may reflect a narrowing performance band among current systems, although the present study cannot establish a true capability ceiling for future architectures.

## Discussion

The longitudinal trajectory of Large Language Model performance on the Chinese National Nurse Licensing Examination from 2022 to 2025 represents a paradigmatic shift in artificial intelligence capabilities within healthcare education. Our comprehensive visual and quantitative analysis (Figs 1–6, Tables 1–3) demonstrates that leading AI systems have transcended the traditional boundaries of pattern recognition to achieve competency levels sufficient for approximate professional nursing credentialing thresholds, yet critical limitations in clinical reasoning and judgment persist, defining the contemporary frontier between artificial intelligence and human clinical expertise.

**Table 3:** Comprehensive error analysis and pattern classification (top 5 models, 2025).

Error Category	Frequency (n)	Percentage	Clinical Significance	Chinese-Native vs International	Representative Examples
**Clinical Reasoning Failures**	619	43.0%	High – Patient safety implications	41.2% vs 45.8% (p = 0.024)	Incorrect intervention sequencing in multi-problem patients; Inappropriate cause-effect reasoning
**Prioritization Failures**	389	27.0%	Critical – Emergency care impact	24.1% vs 31.2% (p < 0.001)	ABC framework misapplication; Failure to identify immediate vs. routine care needs
**Factual Knowledge Deficits**	259	18.0%	Moderate – Knowledge gaps	14.3% vs 23.7% (p < 0.001)	Medication dosage errors; Laboratory value misinterpretation; Protocol misconceptions
**Cultural/Linguistic Errors**	173	12.0%	Variable – Context dependent	3.2% vs 15.8% (p < 0.001)	Chinese medical terminology misunderstanding; Healthcare system-specific practice gaps
**Question Interpretation Errors**	101	7.0%	Low – Technical rather than clinical	6.8% vs 7.3% (p = 0.672)	Misreading question stems; Response option selection mistakes
**Hallucination/Fabrication**	27	1.9%	Very High – Misinformation risk	2.1% vs 1.6% (p = 0.431)	Addition of non-existent patient data; Creation of fictional clinical details
**Total Errors Analyzed**	**1,440**	**100.0%**	—	—	—

Note: Error percentages were calculated from 1,440 incorrect responses among the top 5 performing models (Qwen2.5, ERNIE 4.0, DeepSeek R1, GPT-4o, Claude 3 Opus). Chinese-Native includes Qwen2.5, ERNIE 4.0, and DeepSeek R1. International includes GPT-4o and Claude 3 Opus. Clinical significance ratings were based on potential patient impact assessment by nursing education experts. Some error codes were non-mutually exclusive because prioritization, reasoning, and interpretation failures could co-occur in the same incorrect response; therefore, category percentages should not be summed to 100%.

The remarkable 63% relative improvement in performance among top-tier models, as illustrated in the longitudinal trajectories of Fig 1, reflects the extraordinary pace of AI development during this pivotal period, with annual accuracy gains averaging 10.2 percentage points. This acceleration corresponds directly with fundamental architectural innovations, including the transition from dense transformer architectures to mixture-of-experts systems, the implementation of advanced reasoning frameworks through techniques such as chain-of-thought prompting, and the incorporation of reinforcement learning from human feedback methodologies [24,25]. The consistency of this upward trajectory across diverse model families suggests that current performance levels represent genuine knowledge acquisition rather than examination-specific optimization, indicating that LLMs have developed substantial understanding of nursing principles and practices.

The persistent, non-monotonic performance advantage of Chinese-native models, quantified in Fig 2 and detailed in Table 1, provides empirical evidence for the importance of linguistic and cultural optimization in specialized professional domains. At the top-model level, the gap measured 4.5 percentage points in 2023, 3.0 percentage points in 2024, and 3.8 percentage points in 2025. At the category-mean level, Table 1 shows a narrowing gap from 6.1 percentage points in 2023 to 3.0 percentage points in 2025. Therefore, these findings support a sustained regional advantage without implying a strictly increasing temporal trend in performance differences [26,27]. This phenomenon aligns with recent findings from the MedExamLLM platform, which documented similar regional advantages across multiple medical licensing examinations worldwide [28]. The implications extend beyond mere performance metrics to fundamental questions about healthcare AI deployment strategies, suggesting that future clinical decision support tools, educational resources, and assessment systems may require deep localization to achieve peak effectiveness and cultural relevance.

Our domain-specific analysis, visualized comprehensively in the heatmap of Fig 3, reveals systematic patterns that illuminate both the remarkable capabilities and fundamental limitations of current AI architectures. Interpreted through the Bloom’s taxonomy framework introduced earlier, LLMs demonstrate strongest performance on items requiring knowledge recall and comprehension (lower cognitive levels), while showing consistent deficits on items demanding application, analysis, and evaluative judgment (higher cognitive levels).

The clear performance hierarchy from Fundamentals of Nursing (85.6% accuracy) to Psychiatric Nursing (64.3% accuracy) reflects the core distinction between statistical pattern matching and clinical reasoning [29]. This finding resonates with recent work by Hobensack and colleagues, who identified similar capability gaps in their comprehensive review of LLM applications in nursing practice [19]. The consistent performance differential between Professional Practice and Practical Skills sections, dramatically illustrated in the slope analysis of Fig 4, represents a central finding, highlighting the current boundary between AI as a knowledge repository versus a clinical reasoning partner.

The qualitative error analysis presented in Fig 5 provides crucial insights into failure modes that persist despite overall high-performance levels. The predominance of clinical reasoning failures (43% of errors) and prioritization mistakes (27% of errors) indicates reproducible reasoning vulnerabilities that cannot be explained solely by simple training data deficiencies [30]. The specific difficulty with conditional reasoning and multi-step clinical logic chains indicates that current LLMs can still behave like pattern-sensitive systems when causal reasoning and multi-step prioritization are required, a limitation that has profound implications for autonomous clinical application.

The forest plot analysis in Fig 6, demonstrating clear performance stratification between Chinese-native and international models, reinforces the quantitative findings presented throughout our analysis. The visual clustering of Chinese-native models (red points) in the upper performance tiers versus international models (purple points) in the lower ranges provides compelling evidence for the sustained advantage of regional optimization, even among the most sophisticated AI systems currently available.

The educational implications of these findings, supported by our comprehensive visual analysis, are both promising and concerning. LLMs demonstrate exceptional potential as educational support tools, offering capabilities that could democratize access to high-quality nursing education globally through personalized tutoring, comprehensive knowledge access, and adaptive learning experiences [31,32]. Recent studies have documented successful implementations of AI-powered educational tools in specialized nursing domains, including wound care training and clinical simulation [33]. These recent studies broaden the evidence base beyond wound care and simulation by demonstrating applications in perioperative nursing assessment, specialty-specific surgical question answering, and personalized procedural training [8–10]. However, our identified limitations in clinical reasoning and prioritization, clearly illustrated across multiple analytical approaches, create significant risks if these tools are deployed without appropriate oversight frameworks and critical evaluation protocols [34].

The regulatory and professional implications extend far beyond educational applications to fundamental questions about competency assessment in the era of artificial intelligence. The performance patterns revealed in our analysis suggest that while existing examinations effectively assess knowledge acquisition, they may inadequately evaluate the clinical reasoning, ethical judgment, and communication skills that define safe and effective nursing practice [35]. This concern has been echoed in recent commentaries calling for enhanced evaluation frameworks that better capture the humanistic elements of nursing care [36].

The superior performance of specialized, regionally-optimized models, consistently demonstrated across our analytical framework, has strategic implications for healthcare organizations, technology developers, and regulatory bodies considering AI integration policies. Rather than relying solely on general-purpose systems, future healthcare AI applications may require domain-specific development approaches that incorporate local clinical guidelines, cultural practices, and regulatory frameworks [37,38]. This finding supports emerging trends toward specialized medical AI models, such as Google’s Med-PaLM 2, which achieved expert-level performance through dedicated healthcare training [39].

Our findings contribute to an emerging global discourse on AI capabilities in nursing professional assessment. While the NNLE serves as China’s sole pathway to nursing licensure, analogous examinations exist internationally, including the NCLEX-RN in the United States, the NMC CBT in the United Kingdom, and the HAAD/DHA examinations in the Gulf region. Preliminary evidence suggests that LLM performance patterns observed in our study—specifically, superior performance on knowledge-based items versus clinical reasoning scenarios—may generalize across these international contexts. However, the magnitude of Chinese-native model advantages observed in our study highlights the potential importance of culturally and linguistically adapted AI systems for each regulatory jurisdiction. Future comparative research across international nursing examinations would provide valuable insights into the generalizability of our findings and inform global strategies for AI integration in nursing education and assessment.

Looking toward clinical implementation, our findings position LLMs as powerful augmentative tools rather than autonomous decision-makers. Their exceptional knowledge recall capabilities and comprehensive access to evidence-based guidelines could significantly enhance clinical decision-making when combined with human oversight and critical evaluation [40,41]. However, the persistent limitations in prioritization and complex reasoning, clearly documented across our multi-faceted analysis, mandate careful implementation frameworks that preserve human accountability and clinical judgment primacy. Recent implementations at institutions such as the Mayo Clinic and Johns Hopkins demonstrate successful models for AI integration that maintain appropriate human oversight while leveraging AI capabilities for routine tasks [42].

Future research directions should focus on developing AI systems specifically designed to address the identified limitations in clinical reasoning and prioritization, as revealed through our comprehensive error analysis framework. Advanced training methodologies incorporating explicit causal reasoning frameworks, structured clinical logic chains, and comprehensive ethical decision-making principles may help bridge current capability gaps [43,44]. Additionally, the development of novel assessment frameworks that better evaluate clinical reasoning, communication skills, and ethical judgment will be essential for maintaining the validity and relevance of professional credentialing in the AI era. The emergence of platforms such as the Open Medical-LLM Leaderboard provides valuable infrastructure for ongoing capability assessment and benchmarking [45].

The transformation of nursing education and practice through artificial intelligence integration appears inevitable, but the pace and manner of this change will determine its ultimate benefit or harm to the profession and the patients it serves. As demonstrated by our longitudinal analysis, the capability gap between AI systems and human clinical judgment is narrowing rapidly, but significant domains of uniquely human expertise persist. Maintaining focus on these irreplaceable elements—clinical intuition, therapeutic presence, ethical reasoning, and holistic care coordination—will be essential for preserving the integrity and effectiveness of professional nursing practice while harnessing the transformative potential of artificial intelligence.

## Conclusion

This comprehensive longitudinal analysis demonstrates that Large Language Models have achieved remarkable proficiency in nursing knowledge domains, with leading systems exceeding the approximate raw-score benchmark associated with the Chinese National Nurse Licensing Examination by 2025. However, significant deficiencies in clinical reasoning, particularly prioritization skills and complex judgment scenarios, define the current boundary between artificial intelligence performance on written examinations and human clinical expertise. Critically, examination performance should not be interpreted as evidence of clinical competence or readiness for autonomous patient care, as 43% of errors among top-performing models involved clinical reasoning failures and 27% involved prioritization errors, precisely the capabilities most essential for safe patient care.

The observed advantages of regionally-optimized models underscore the importance of cultural and linguistic adaptation for specialized healthcare applications. For nursing education and practice, these findings present both opportunities and imperatives: AI systems offer promising tools for personalized learning support and knowledge access, yet persistent limitations in clinical reasoning reinforce the irreplaceable value of human nursing expertise. As the profession navigates this technological transformation, nursing curricula should increasingly emphasize higher-order cognitive and interpersonal competencies that distinguish expert human practice from artificial intelligence capabilities.

For regulatory bodies and healthcare institutions, we recommend implementing AI-powered educational tools as supplements to human instruction, incorporating examination items that assess capabilities challenging for current AI systems, and establishing robust governance frameworks that preserve human accountability for clinical decision-making while leveraging AI capabilities for knowledge support.

## Supporting information

S1 File Supplementary Materials. (DOCX)
