# Supplementary material for: From knowledge to judgment: A three-year longitudinal analysis of artificial intelligence large language model performance on the Chinese national nurse licensing examination
Source: PLoS One. 2026 Jul 2;21(7):e0353059. doi: 10.1371/journal.pone.0353059 (PMC13327122; doi:10.1371/journal.pone.0353059)
Supplement: S1 File — (DOCX) [file pone.0353059.s001.docx]

**Human Participants Research Checklist**

***Complete the following if your study involved human participants or human participants’ data. These questions should be addressed for prospective and retrospective studies.***

1. Did you obtain ethics approval for this study?
   - If yes, please upload (file type “Other”) all the approval documents you received from your ethics committee to cover the entire range of the study period (i.e. the original approval document and any extension documents). Where ethics approval was obtained from more than one study location, please provide approval document(s) from all of the sites. If the original document is in another language, please also provide an English translation.

**N/A**

- If you did not obtain ethical approval, please explain why this was not required below.

This computational study involved no human participants. The research evaluated artificial intelligence language models using publicly available examination materials only. Our Institutional Research Ethics Committee at Sir Run Run Shaw Hospital, Zhejiang University School of Medicine granted exemption status (Protocol #2024-NURS-089) specifically because no human subjects were involved. No approval documents are applicable as this study falls outside human subjects research requirements."

2. If you prospectively recruited human participants for the study – for example, you conducted a clinical trial, distributed questionnaires, or obtained tissues, data or samples for the purposes of this study, please report in the Methods:
   i. the day, month and year of the **start and end** of the recruitment period for this study.
   ii. whether participants provided informed consent, and if so, what type was obtained (for instance, written or verbal, and if verbal, how it was documented and witnessed). If your study included minors, state whether you obtained consent from parents or guardians. If the need for consent was waived by the ethics committee, please include this information.

**Response:** No human participants were prospectively recruited. This study exclusively evaluated artificial intelligence language models (GPT-4, Claude, ERNIE, Qwen, etc.) using automated computational methods. No humans were recruited, surveyed, tested, or had samples/data collected for research purposes."

Please state the line number(s) in the Methods where this is reported: **N/A**

3. If you are reporting a retrospective study of, for example, medical records, archived samples, survey data, please report in the Methods section:
i. the day, month and year when the data were accessed for research purposes
ii. whether authors had access to information that could identify individual participants during or after data collection

Please state the line number(s) in the Methods where this is reported: **N/A**

**Response:** This was not a retrospective study of human data. No medical records, archived human samples, or survey data were accessed. The study used only publicly available educational examination questions from the Chinese National Nurse Licensing Examination, which are published training materials rather than human research data."
