# Supplementary material for: From knowledge to judgment: A three-year longitudinal analysis of artificial intelligence large language model performance on the Chinese national nurse licensing examination
Source: PLoS One. 2026 Jul 2;21(7):e0353059. doi: 10.1371/journal.pone.0353059 (PMC13327122; doi:10.1371/journal.pone.0353059)
Supplement: Attachment — Submitted filename: PLOSOne_Human_Subjects_Research_Checklist.docx [file pone.0353059.s002.docx]

**Response to Reviewers**

**Manuscript ID:** PONE-D-25-46495

**Title:** From Knowledge to Judgment: Tracking LLM Progress on the Chinese National Nurse Licensing Examination Over Three Years

**Dr. Krit Pongpirul, MD, MPH, PhD.**

Academic Editor PLOS ONE

Dear Dr. Pongpirul,

We wish to express our sincere gratitude to you and the distinguished reviewers for the thorough and constructive evaluation of our manuscript. The insightful comments have been invaluable in strengthening the scientific rigor and clarity of our work. We have carefully considered each point raised and have undertaken substantial revisions to address all concerns comprehensively.

Below, we provide detailed responses to each comment from both reviewers, along with descriptions of the corresponding modifications made to the manuscript. All changes have been tracked in the revised manuscript for ease of identification.

**RESPONSE TO JOURNAL REQUIREMENTS**

**1. *Please ensure that your manuscript meets PLOS ONE's style requirements, including those for file naming. The PLOS ONE style templates can be found at***

***https://journals.plos.org/plosone/s/file?id=wjVg/PLOSOne_formatting_sample_main_body.pdf and***

[***https://journals.plos.org/plosone/s/file?id=ba62/PLOSOne_formatting_sample_title_authors_affiliations.pdf***](https://track.editorialmanager.com/CL0/https:%2F%2Fjournals.plos.org%2Fplosone%2Fs%2Ffile%3Fid=ba62%2FPLOSOne_formatting_sample_title_authors_affiliations.pdf/1/010f019ab999ac76-1ccd6714-7d70-4e81-83ff-47f83e6bcf9b-000000/vD12JV1tu6zrdPCNXEicykqYNc8Azi10TOYbbVkDiIU=238)

**Response:** We have carefully reviewed the PLOS ONE style templates and reformatted the manuscript to ensure full compliance with the journal's formatting guidelines, including file naming conventions, heading structures, and reference formatting. Additionally, all references throughout the manuscript have been updated and arranged according to PLOS ONE's reference style requirements.

***2. Please note that PLOS One has specific guidelines on code sharing for submissions in which author-generated code underpins the findings in the manuscript. In these cases, we expect all author-generated code to be made available without restrictions upon publication of the work. Please review our guidelines at https://journals.plos.org/plosone/s/materials-and-software-sharing#loc-sharing-code and ensure that your code is shared in a way that follows best practice and facilitates reproducibility and reuse.***

**Response:** We acknowledge the journal's guidelines on code sharing. As our study involved API-based evaluation of commercial and open-source language models rather than custom-developed code, we have clarified in the revised manuscript that no proprietary code was developed for this research. The standardized prompting protocols and evaluation procedures have been fully described in the Methods section to ensure complete reproducibility.

***3. Please include your tables as part of your main manuscript and remove the individual files. Please note that supplementary tables (should remain/ be uploaded) as separate "supporting information" files.***

**Response:** We have integrated Tables 1-3 directly into the main manuscript body at appropriate locations following their first textual reference, as requested. The tables have been removed as separate files.

**4. *Thank you for stating the following financial disclosure:***

***[This work was supported by the Zhejiang Provincial Natural Science Foundation of China (Grant No. LQ24H160021) and Zhejiang medical and health project (Grant No. 2023KY781).].***

***Please state what role the funders took in the study.  If the funders had no role, please state: "The funders had no role in study design, data collection and analysis, decision to publish, or preparation of the manuscript."***

***If this statement is not correct you must amend it as needed.***

***Please include this amended Role of Funder statement in your cover letter; we will change the online submission form on your behalf.***

**Response:** We sincerely apologize for this discrepancy. The funding statement in the original manuscript was erroneous. This work was indeed supported by the Zhejiang Provincial Natural Science Foundation of China (Grant No. LQ24H160021) and Zhejiang Medical and Health Project (Grant No. 2023KY781). We have corrected the Funding section in the revised manuscript to accurately reflect this support. Regarding the role of funders: **"The funders had no role in study design, data collection and analysis, decision to publish, or preparation of the manuscript."**

**5. *In the online submission form, you indicated that [Available from the corresponding author upon reasonable request.].***

***All PLOS journals now require all data underlying the findings described in their manuscript to be freely available to other researchers, either 1. In a public repository, 2. Within the manuscript itself, or 3. Uploaded as supplementary information.***

***This policy applies to all data except where public deposition would breach compliance with the protocol approved by your research ethics board. If your data cannot be made publicly available for ethical or legal reasons (e.g., public availability would compromise patient privacy), please explain your reasons on resubmission and your exemption request will be escalated for approval.***

**Response:** We acknowledge that our previous data availability statement required clarification regarding PLOS ONE's data policy. The data underlying this study are subject to institutional data governance policies at Sir Run Run Shaw Hospital, Zhejiang University School of Medicine, which restrict public dissemination of research datasets without prior institutional approval. This policy exists to ensure appropriate oversight of data sharing and compliance with institutional research governance standards.

Therefore, we respectfully request an exemption from the standard data availability requirements based on institutional policy restrictions. To ensure transparency and facilitate reproducibility within these constraints, we have revised our Data Availability Statement to read: **"The data underlying this study are subject to institutional data governance policies at Sir Run Run Shaw Hospital, Zhejiang University School of Medicine. Data requests may be sent to the institutional data contact: Ms. Jiang Miaomiao (jiang0304@zju.edu.cn), Nursing Department, Sir Run Run Shaw Hospital, Zhejiang University School of Medicine. Qualified researchers may access data subject to institutional approval and completion of a data sharing agreement. Researchers may also contact the corresponding author, Dr. Jionghuang Chen (zxj6682@126.com), for coordination of data access requests. The institutional repository ensures long-term data preservation per the hospital's 10-year research data retention policy."**

This approach balances PLOS ONE's commitment to transparency with our institutional obligations.

**6. *PLOS requires an ORCID iD for the corresponding author in Editorial Manager on papers submitted after December 6th, 2016. Please ensure that you have an ORCID iD and that it is validated in Editorial Manager. To do this, go to ‘Update my Information’ (in the upper left-hand corner of the main menu), and click on the Fetch/Validate link next to the ORCID field. This will take you to the ORCID site and allow you to create a new iD or authenticate a pre-existing iD in Editorial Manager.***

**Response:** The corresponding author has validated the ORCID iD: 0009-0007-2618-6500 in Editorial Manager as requested.

**7. *Please include a copy of Table 1-3 which you refer to in your text on page 22.***

**Response:** Tables 1-3 are now included within the main manuscript text at the locations where they are first referenced.

**8. *If the reviewer comments include a recommendation to cite specific previously published works, please review and evaluate these publications to determine whether they are relevant and should be cited. There is no requirement to cite these works unless the editor has indicated otherwise.***

**Response:** We have carefully evaluated all citations recommended by the reviewers and incorporated those deemed relevant and scientifically appropriate into the revised manuscript.

**RESPONSE TO REVIEWER #1**

We are deeply appreciative of Reviewer #1's meticulous examination of our manuscript. The reviewer has identified several critical issues that required immediate attention, and we have addressed each concern with the utmost seriousness.

**Comment 1: Funding Statement Contradiction**

*The manuscript declares in the "Funding" section (line 504) that "This research received no specific grant funding," yet the submission form explicitly states it was supported by two Chinese grants.*

**Response:** We sincerely apologize for this significant oversight, which resulted from an administrative error during manuscript preparation. The correct information is that this research was supported by the Zhejiang Provincial Natural Science Foundation of China (Grant No. LQ24H160021) and Zhejiang Medical and Health Project (Grant No. 2023KY781). We have corrected the Funding section to read: **"This work was supported by the Zhejiang Provincial Natural Science Foundation of China (Grant No. LQ24H160021) and the Zhejiang Medical and Health Project (Grant No. 2023KY781). The funders had no role in study design, data collection and analysis, decision to publish, or preparation of the manuscript."**

**Comment 2: Inadequate Data Availability Compliance**

*The manuscript's Data Availability statement reads: "Available from the corresponding author upon reasonable request." This phrasing violates PLOS ONE's data policy.*

**Response:** We appreciate the reviewer highlighting this important issue and wish to provide clarification. The data underlying this study are subject to institutional data governance policies at Sir Run Run Shaw Hospital, Zhejiang University School of Medicine, which restrict public dissemination of research datasets without prior institutional approval. This institutional policy exists to ensure appropriate oversight of data sharing and compliance with research governance standards.

We have revised the Data Availability Statement in the Declarations section to transparently explain this restriction: **"The data underlying this study are subject to institutional data governance policies at Sir Run Run Shaw Hospital, Zhejiang University School of Medicine. Data requests may be sent to the institutional data contact: Ms. Jiang Miaomiao (jiang0304@zju.edu.cn), Nursing Department, Sir Run Run Shaw Hospital, Zhejiang University School of Medicine. Qualified researchers may access data subject to institutional approval and completion of a data sharing agreement. Researchers may also contact the corresponding author, Dr. Jionghuang Chen (zxj6682@126.com), for coordination of data access requests. The institutional repository ensures long-term data preservation per the hospital's 10-year research data retention policy.""**

This revised statement complies with PLOS ONE's policy for cases where institutional restrictions prevent full public data sharing, as it clearly specifies the nature of the restriction and provides a clear pathway for qualified researchers to request data access through appropriate channels.

**Comment 3: Authorship and Contributor Mismatch**

*The "Authors' Contributions" section lists individuals with initials "T-TY" and "X-LH" (lines 507-508), but these do not correspond to any of the four named authors.*

**Response:** We are grateful for identifying this serious error, which occurred due to a copy-paste mistake during manuscript compilation from an earlier draft. We have thoroughly revised the Authors' Contributions section to accurately reflect the contributions of the four named authors: **"X-JZ and J-HC contributed equally to study design, data collection, and manuscript preparation. J-SC performed statistical analyses and figure preparation. W-HY supervised the study and provided critical manuscript revisions. All authors read and approved the final manuscript."**

**Comment 4: Ethics Statement Inconsistency**

*The online submission form marks the Ethics Statement as "N/A," but the manuscript describes IRB exemption (#2024-NURS-089).*

**Response:** We acknowledge this inconsistency and have rectified the submission form to accurately reflect our IRB exemption status. The study appropriately received exemption from the Institutional Research Ethics Committee at Sir Run Run Shaw Hospital (Protocol #2024-NURS-089) as it involved only publicly available educational materials and computational model evaluation with no human subjects. We have updated the online submission form accordingly.

**Comment 5: Unsubstantiated Passing Threshold Claim**

*The authors equate 60% raw accuracy with passing the Chinese National Nurse Licensing Examination (which uses a 300-point scaled score). However, they provide no evidence linking raw percentage correct to the official scaled passing standard.*

**Response:** We appreciate this important methodological concern. We have substantially revised our discussion of the passing threshold to address this limitation transparently. The revised manuscript now includes the following clarification in the Methods section: **"It should be noted that the NNLE employs a scaled scoring system with a passing threshold of 300 points. While the precise algorithm for converting raw scores to scaled scores is not publicly disclosed by the National Medical Examination Center, historical analysis of examination outcomes and preparatory materials consistently indicates that approximately 60% raw accuracy corresponds to passing performance. We acknowledge this as a methodological limitation and have adopted this threshold as an approximate benchmark rather than an exact equivalence."** Additionally, we have moderated our claims regarding "passing" performance throughout the manuscript to reflect this uncertainty.

**Comment 6: Ambiguous Temporal Evaluation Protocol**

*While the study claims "temporal fidelity," it lacks specifics: Which exact model versions were used? Were mid-year updates accounted for?*

**Response:** We thank the reviewer for highlighting this important gap in methodological transparency. We have added comprehensive supplementary materials detailing the exact model versions, API endpoints, and access dates for each evaluation. Supplementary Table S1 provides complete model version documentation for all international and Chinese-native LLMs, including specific API endpoints, parameter counts, release dates, evaluation periods, and training data cutoffs. Supplementary Table S2 documents the standardized inference parameters, and Supplementary Table S3 details the hardware and software specifications for local inference of open-source models. Furthermore, we have expanded the Methods section to include: **"To ensure temporal fidelity, we documented specific model versions and API endpoints for each evaluation period. For models with mid-year updates (e.g., GPT-4 to GPT-4-turbo), we utilized the version available at the time of each examination cycle's evaluation. Specifically, GPT-4 evaluations in 2023 employed the gpt-4-0314 endpoint, while 2024 evaluations utilized gpt-4-0613. Complete version documentation is provided in Supplementary Table S1-S3."**

**Comment 7: Overstatement of Clinical Competence**

*Phrases like "sufficient codified nursing knowledge to pass professional licensing examinations" may imply clinical readiness. The conclusion should more clearly emphasize that exam performance ≠ safe clinical judgment.*

**Response:** We concur entirely with this concern and have substantially revised our language throughout the manuscript to avoid any implication of clinical readiness. The revised Conclusion now emphasizes: **"Critically, the ability to pass a professional licensing examination should not be interpreted as evidence of clinical competence or readiness for autonomous patient care. Our error analysis reveals that 43% of mistakes among top-performing models involved clinical reasoning failures and 27% involved prioritization errors—precisely the capabilities most essential for safe patient care. These findings underscore that current LLMs function as sophisticated knowledge repositories rather than clinical reasoning agents, and any deployment in healthcare settings must incorporate robust human oversight frameworks."**

**Comment 8: Missing Inference Protocol Details**

*The evaluation used "standardized zero-shot prompting," but key parameters (e.g., temperature, top-p, max tokens, API vs. local inference) are not reported.*

**Response:** We appreciate this crucial observation regarding reproducibility. We have expanded the Methods section to include complete inference parameters: **"All API-based evaluations employed the following standardized parameters: temperature = 0.0 to ensure deterministic outputs, top_p = 1.0, max_tokens = 100, and no system prompts beyond the standardized question format. For open-source models (Llama series), inference was conducted using the Hugging Face Transformers library (version 4.35.0) with identical temperature and sampling parameters on NVIDIA A100 GPUs with 80GB memory. Response parsing utilized regular expression matching to extract single-letter answers, with manual review of any responses not conforming to the expected format."**

**Comment 9: Unclear Copyright and Redistribution Status**

*It is unclear whether the compiled 7,200-question dataset can be legally shared.*

**Response:** We appreciate the reviewer raising this important concern. Upon thorough review of the copyright status of our examination corpus, we confirm that the questions are derived from officially published preparatory materials of the National Medical Examination Center (NMEC) of China. These materials are protected under copyright, and public redistribution is not permitted under the terms of their use.

Accordingly, we have clarified this in the revised manuscript: **"The examination corpus was compiled from officially endorsed preparatory materials distributed through the China Health Human Resources portal (**[**www.21wecan.com**](http://www.21wecan.com)**). These materials are protected under copyright held by the National Medical Examination Center of China, and public redistribution is not permitted. Researchers wishing to replicate this study may independently obtain examination materials through the official portal."**

This approach ensures transparency while respecting intellectual property rights. Our analytical data (model responses, accuracy calculations, and error classifications) can be made available upon reasonable request to facilitate verification of our findings.

**RESPONSE TO REVIEWER #2**

We are grateful for Reviewer #2's thoughtful and constructive feedback, which has helped us strengthen the theoretical foundations and practical implications of our work.

**Comment 1: Theoretical Framework Clarification**

*The introduction section requires a thorough clarification of theoretical framework of the study.*

**Response:** We have substantially expanded the Introduction to articulate a clear theoretical framework, integrating it naturally within the existing narrative structure rather than presenting it as a discrete section. Specifically, we have woven the theoretical underpinnings into two locations where they contextually enhance the scientific rationale. First, when discussing nursing competencies and the demands of the profession, we introduce Bloom's revised taxonomy to frame how nursing competence can be conceptualized hierarchically, from foundational knowledge recall through higher-order evaluative judgment, drawing upon recent systematic reviews examining AI applications in nursing education (Bozkurt, 2025). Second, when discussing the research gaps and our study's contribution, we incorporate contemporary skill acquisition theory to position LLM capabilities relative to human professional development stages, referencing recent work applying these frameworks to AI in nursing contexts (Goktas et al., 2024). This integrated approach ensures the theoretical framework enhances rather than interrupts the logical flow of the Introduction, providing readers with the conceptual tools necessary to interpret our findings while maintaining narrative coherence.

**Comment 2: AI Systems Prominence in Title**

*The AI systems are mentioned in many parts of the research but not prominent in the title.*

**Response:** We appreciate this observation regarding title clarity. After careful consideration, we propose revising the title to more explicitly reference AI systems: **"From Knowledge to Judgment: A Three-Year Longitudinal Analysis of Artificial Intelligence Large Language Model Performance on the Chinese National Nurse Licensing Examination."** This revision maintains the conceptual framing while making the AI focus more prominent. We welcome the editor's guidance if an alternative formulation would be preferred.

**Comment 3: Chinese-Native LLM Examples (Lines 97-98)**

*More clarification or definition is needed for the examples of Chinese-native LLMs.*

**Response:** We have expanded this section to provide clearer definitions and examples: **"Chinese-native LLMs refer to large language models developed by Chinese technology companies with primary optimization for Chinese language understanding and generation. These include Baidu's ERNIE (Enhanced Representation through kNowledge IntEgration) series, which incorporates structured knowledge graphs and Chinese linguistic features; Alibaba's Qwen (Tongyi Qianwen) models, designed with extensive Chinese web corpus training; Tencent's Hunyuan series, optimized for Chinese conversational contexts; and DeepSeek's reasoning-focused models, which employ novel architectural approaches to enhance logical inference capabilities. These models differ from international counterparts in their training data composition, tokenization strategies optimized for Chinese characters, and incorporation of Chinese cultural and linguistic nuances."**

**Comment 4: Domain Clarification (Lines 144-146)**

*Clarify if these domains are the only official domains studied in Chinese nursing institutions, if not, why other domains as nursing administration or critical care nursing are not included in the exam and the study.*

**Response:** We have added clarification regarding the examination structure: **"The six content domains evaluated in this study—Fundamentals of Nursing, Medical-Surgical Nursing, Obstetrics/Gynecology Nursing, Pediatric Nursing, Psychiatric Nursing, and Ethics & Regulations—represent the official content specifications established by the National Medical Examination Center for the NNLE. While Chinese nursing education encompasses additional specialized areas such as nursing administration, critical care nursing, and community health nursing, these domains are not separately assessed in the national licensing examination. The NNLE is designed to evaluate entry-level competency for general nursing practice, with specialized competencies assessed through separate certification processes following licensure."**

**Comment 5: Zero-Shot Prompting Example (Line 166)**

*Kindly give example to clearly define the concept of zero-shot prompting protocols.*

**Response:** We have expanded our explanation of zero-shot prompting with a concrete example: **"Zero-shot prompting refers to presenting questions to the model without providing any example questions and answers (demonstrations) beforehand, requiring the model to respond based solely on its pre-existing knowledge. In contrast to few-shot prompting, which provides several examples to guide response formatting, zero-shot evaluation more accurately reflects real-world deployment scenarios where models must generalize without task-specific examples. Our standardized prompt was: '请仔细阅读以下护理执业资格考试题目，并从A、B、C、D四个选项中选择最正确的答案。请只回答选项字母。[Question text and options]' (Please carefully read the following nursing licensure examination question and select the most correct answer from options A, B, C, D. Please respond only with the option letter. [Question text and options]). This approach ensures that observed performance reflects the models' inherent capabilities rather than their ability to pattern-match from provided examples."**

**Comment 6: Results Section**

*In regard to the results section, it was concise, clear and to the point.*

**Response:** We thank the reviewer for this positive assessment and have maintained the structure of the Results section while incorporating minor refinements for enhanced clarity.

**Comment 7: International Nursing Examination Perspective**

*It will be better to add a researchers' point of view regarding the nursing licensing examinations internationally at the end.*

**Response:** We have added a new paragraph to the Discussion section providing international context: **"Our findings contribute to an emerging global discourse on AI capabilities in nursing professional assessment. While the NNLE serves as China's sole pathway to nursing licensure, analogous examinations exist internationally, including the NCLEX-RN in the United States, the NMC CBT in the United Kingdom, and the HAAD/DHA examinations in the Gulf region. Preliminary evidence suggests that LLM performance patterns observed in our study—specifically, superior performance on knowledge-based items versus clinical reasoning scenarios—may generalize across these international contexts. However, the magnitude of Chinese-native model advantages observed in our study highlights the potential importance of culturally and linguistically adapted AI systems for each regulatory jurisdiction. Future comparative research across international nursing examinations would provide valuable insights into the generalizability of our findings and inform global strategies for AI integration in nursing education and assessment."**

**Comment 8: Lines 457-461 Relocation**

*I think it would be good to transfer these lines to the conclusion and practical recommendation sections.*

**Response:** We have relocated the content from lines 457-461 to the Conclusion section and expanded upon the practical recommendations: **"For nursing educators and regulatory bodies, our findings suggest several actionable recommendations. First, AI-powered educational tools should be implemented as supplements to, rather than replacements for, human instruction, particularly for developing clinical reasoning and prioritization skills. Second, examination frameworks may benefit from incorporating items specifically designed to assess capabilities that current AI systems find challenging, thereby maintaining discriminant validity between human and artificial test-takers. Third, healthcare institutions considering AI integration should implement robust governance frameworks that preserve human accountability for clinical decision-making while leveraging AI capabilities for knowledge support and routine information processing."**

**Comment 9: Conclusion Clarification for Nursing Profession**

*The conclusion needs clarification for the nursing profession and for the nursing practice.*

**Response:** We have revised the Conclusion to provide more explicit and concise guidance for nursing professionals and practice while maintaining strength and clarity. The revised conclusion now includes clear implications for the nursing profession, noting that AI systems present opportunities for personalized learning support and knowledge access, while persistent limitations in clinical reasoning reinforce the irreplaceable value of human nursing expertise. We emphasize that nursing curricula should increasingly emphasize higher-order cognitive and interpersonal competencies. Additionally, we provide specific, actionable recommendations for regulatory bodies and healthcare institutions regarding AI-powered educational tools, examination design, and governance frameworks—all presented in a streamlined format that enhances readability and impact.
