# Supplementary material for: From knowledge to judgment: A three-year longitudinal analysis of artificial intelligence large language model performance on the Chinese national nurse licensing examination
Source: PLoS One. 2026 Jul 2;21(7):e0353059. doi: 10.1371/journal.pone.0353059 (PMC13327122; doi:10.1371/journal.pone.0353059)
Supplement: Attachment — Submitted filename: Response Letter.docx [file pone.0353059.s003.docx]

Response to the Academic Editor and Reviewers

Dear Dr. Amankwaa,

On behalf of all authors, I sincerely thank you and the reviewers for the careful re-evaluation of our manuscript and for the constructive guidance provided in this revision round. We have revised the manuscript with focused attention to numerical consistency, corpus accounting, reproducibility, data leakage safeguards, statistical transparency, error-coding reliability, figure and table clarity, and the ethical interpretation of artificial intelligence performance in nursing contexts. We also confirm that Supplementary Tables S1-S3 are submitted as a separate Supporting Information file.

For transparency, the editor and reviewer comments are reproduced below using the same wording as presented in the decision letter. Where Reviewer 3 provided a consolidated list of issues, we separated the list into individual response items while preserving the reviewer’s wording. The line references below refer to the clean revised manuscript with line numbering. Because line numbering can shift after Editorial Manager regenerates the submission PDF, we also identify the relevant manuscript section, table, or figure legend for each change.

In preparing the final submission package, we rebuilt the manuscript from the original Word file so that the EndNote citation field structure remained intact. This response letter is a standalone document and does not contain EndNote field codes.

# Response to the Academic Editor

**Academic Editor Comment 1.** *Reconcile contradictory performance-gap figures. The abstract states that the Chinese-native versus international performance gap increased from 1.6% (2023) to 3.8% (2025), and the Discussion repeats this. Table 1 reports performance gaps of 6.1% (2023), 4.2% (2024), and 3.0% (2025) — a decreasing gap. The Results body separately notes a peak of 4.5 percentage points in 2023. Please clarify which figures are correct and revise the abstract, results narrative, discussion, and cover letter so that they tell a single consistent story. If the direction of the gap is genuinely changing over time, the abstract claim of an "increasing" gap is contradicted by Table 1.*

**Response.** We appreciate this careful observation and agree that the previous revision unintentionally conflated two different gap definitions. After rechecking the calculations, we clarified that Table 1 reports the mean category-level difference between Chinese-native models and international models, with gaps of 6.1, 4.2, and 3.0 percentage points from 2023 to 2025. Figure 2 and the relevant Results narrative report the top-model gap, defined as the difference between the single highest-performing Chinese-native model and the single highest-performing international model in each year, with values of 4.5, 3.0, and 3.8 percentage points from 2023 to 2025. We therefore removed the prior statement that the gap increased over time and revised the Abstract, Results, Discussion, Table 1 note, and Figure 2 legend so that the manuscript presents one coherent interpretation: the mean category-level advantage decreased over time, while the top-model advantage remained persistent but non-monotonic. These revisions appear in the Abstract Results paragraph at lines 48-58, the Table 1 note at lines 327-330, the Comparative Analysis subsection at lines 332-343, the Discussion paragraph on regional optimization at lines 512-524, and the revised Figure 2 legend.

**Academic Editor Comment 2.** *Correct the section-level accuracy figures in the abstract. The abstract reports Professional Practice = 83.5% and Practical Skills = 74.6%. The Results body and Table 2 indicate Professional Practice = 81.6% and Practical Skills = 70.9%; the 83.5% figure corresponds to the Ethics & Regulations domain rather than the Professional Practice section. Please correct the abstract to use the section-level figures consistent with the Results.*

**Response.** We agree and have corrected the Abstract to match Table 2 and the Results body. The Professional Practice section is now reported as 81.6% average accuracy, and the Practical Skills section is now reported as 70.9% average accuracy. The 83.5% value is retained only for the Ethics & Regulations domain, where it belongs. This correction appears in the Abstract Results paragraph at lines 54-56 and is consistent with the Section-Specific Performance Patterns subsection at lines 393-404 and Table 2.

**Academic Editor Comment 3.** *Confirm Supplementary Tables S1–S3 are uploaded. Your response to Reviewer #1 Comments 6 and 8 indicates that Supplementary Tables S1 (model versions, API endpoints, training data cutoffs), S2 (inference parameters), and S3 (hardware/software specifications) have been added. These are referenced in the Methods but do not appear in the merged submission file I reviewed. Please confirm they are attached as Supporting Information.*

**Response.** We confirm that Supplementary Tables S1, S2, and S3 are included in the separate Supporting Information file submitted with this revision. To make this explicit for readers and editorial staff, we revised the Technical Specifications paragraph to state that complete version documentation, API endpoints, parameter counts, training data cutoffs, inference parameters, and hardware/software specifications are provided in Supplementary Tables S1-S3 and submitted separately as Supporting Information. This clarification appears at lines 186-196. The separate supplementary file contains Tables S1-S3 in full.

**Academic Editor Comment 4.** *Reconcile the corpus arithmetic with the study span. The Methods state that the corpus spans July 2022 to June 2025 and contains 7,200 questions, with a breakdown of 2,200 (2022), 2,400 (2023), and 2,600 (2024). The 2025 examination cycle is missing from this breakdown despite featuring centrally in Table 1 and throughout the Results. Please either add the 2025 question count or clarify the corpus composition.*

**Response.** We agree that the previous corpus description was incomplete. We corrected the Methods and Abstract to state that the validated corpus comprised 9,800 questions across four examination years: 2,200 from 2022, 2,400 from 2023, and 2600 questions in each of 2024 and 2025. This correction aligns the corpus description with the 2025 analyses reported in Tables 1 and 2 and throughout the Results. The Abstract Methods paragraph was revised at lines 40-47, and the Final Corpus Characteristics paragraph was revised at lines 166-179.

# Response to Journal Requirements

**Journal Requirement 1.** *If the reviewer comments include a recommendation to cite specific previously published works, please review and evaluate these publications to determine whether they are relevant and should be cited. There is no requirement to cite these works unless the editor has indicated otherwise.*

**Response.** We reviewed and evaluated the papers suggested by Reviewer 3 under this guidance. Because the suggested works are broad or preprint-stage contributions and are not all directly focused on nursing licensure examinations, the Chinese NNLE, or clinical AI governance in nursing practice, we did not add them automatically. Instead, we retained a focused reference list centered on nursing education, medical examination performance, clinical AI evaluation, and healthcare AI governance, while revising the manuscript language to acknowledge rapidly evolving work on uncertainty-aware evaluation, alignment, instruction following, and model adaptation. This approach maintains the manuscript’s disciplinary focus while addressing the reviewer’s concern that the field is advancing quickly.

**Journal Requirement 2.** *Please review your reference list to ensure that it is complete and correct. If you have cited papers that have been retracted, please include the rationale for doing so in the manuscript text, or remove these references and replace them with relevant current references. Any changes to the reference list should be mentioned in the rebuttal letter that accompanies your revised manuscript. If you need to cite a retracted article, indicate the article’s retracted status in the References list and also include a citation and full reference for the retraction notice.*

**Response.** We reviewed the reference list for completeness, formatting consistency, and relevance to the revised manuscript. We did not intentionally cite any retracted work. No retracted article is required for the argument of the manuscript, and no retraction notice needed to be added. The response to Reviewer 3 below explains how we evaluated the suggested recent literature and why the final reference list remains focused on sources most directly relevant to nursing licensure, healthcare AI assessment, and clinical governance.

# Response to Reviewer 2

**Reviewer 2 Comment.** *All comments have been addressed*

**Response.** We sincerely thank Reviewer 2 for confirming that all previous comments have been addressed and for affirming that the manuscript is technically sound, statistically appropriate, supported by available data, and written in clear English. We made no additional changes specifically in response to Reviewer 2 beyond the final editorial corrections, consistency checks, and response-letter refinements described in this document.

# Response to Reviewer 3

Reviewer 3 provided a consolidated list of major issues, minor issues, and a related-work suggestion. We respond to each point individually below, preserving the wording of the reviewer’s comments while explaining the exact revisions made in the manuscript.

## Major Issues

**Reviewer 3 Major Issue 1.** *Methods insufficiently described — Dataset source, prompting strategy, and reproducibility details are unclear.*

**Response.** We thank the reviewer for identifying areas where the Methods needed greater precision. We expanded the Methods without changing the manuscript structure. The Data Source Identification and Final Corpus Characteristics sections now give a corrected corpus count and year-level distribution. The Technical Specifications paragraph now explicitly directs readers to Supplementary Tables S1-S3 for model versions, API endpoints, parameter counts, training data cutoffs, inference parameters, and hardware/software specifications. The prompting strategy remains zero-shot, but the Evaluation Protocol section now more clearly explains the standardized Chinese prompt, response extraction, and deterministic inference settings. These changes appear at lines 166-196 and lines 203-215. Additional reproducibility details, including the temporal fidelity and data leakage safeguards, appear at lines 223-233.

**Reviewer 3 Major Issue 2.** *Possible data leakage not adequately addressed.*

**Response.** We agree that data leakage is a critical concern in evaluations of contemporary LLMs, particularly because the full training corpora of proprietary and open-weight models are not completely transparent. We revised the Methods to state this limitation explicitly and to explain the safeguards used in the study. These safeguards included evaluating models only under the specified temporal fidelity protocol, documenting model version strings and training data cutoffs where available, screening questions for unstable or unattributable source material, and checking for duplicate or verbatim publicly indexed items where feasible. We also clarified that residual exposure cannot be eliminated with absolute certainty and should be considered a limitation of the study. These revisions appear at lines 223-233.

**Reviewer 3 Major Issue 3.** *Overinterpretation of results — Exam performance should not be equated with clinical competence.*

**Response.** We fully agree with this concern and strengthened the manuscript accordingly. The Abstract, Discussion, and Conclusion now use more careful language that frames model output as written examination performance rather than clinical competence, clinical readiness, or autonomous practice capability. We also softened deterministic statements about architecture and performance ceilings. For example, the Practical Skills interpretation now states that the study cannot determine whether the observed application gap arises primarily from architecture, training data, prompting, item type, or examination structure. This revision appears at lines 398-406. The Discussion of error patterns was similarly tempered at lines 539-546. The revised Conclusion states explicitly that written examination performance should not be interpreted as evidence of clinical competence or readiness for autonomous patient care, as shown at lines 617-628.

**Reviewer 3 Major Issue 4.** *Statistical methods need clarification (assumptions, multiple comparisons).*

**Response.** We revised the Statistical Testing Procedures paragraph to clarify the analytical assumptions and correction procedures. The revised text now specifies residual diagnostics, Shapiro-Wilk testing for normality, Levene testing for homogeneity of variance, the use of Welch or nonparametric sensitivity analyses when assumptions were not met, and Bonferroni correction for multiple comparisons. These changes appear at lines 250-259 and strengthen the reproducibility and interpretability of the reported statistical comparisons.

**Reviewer 3 Major Issue 5.** *Error analysis is strong but requires explanation of coding procedures and reviewer reliability.*

**Response.** We appreciate the reviewer’s positive assessment of the error analysis and agree that the coding procedures required clearer reporting. We revised the Qualitative Analysis Framework to describe the a priori codebook, pilot coding of 100 incorrect responses, independent dual coding, consensus resolution, and reviewer reliability. Cohen’s kappa before consensus was κ = 0.86, indicating strong agreement. We also clarified in the Table 3 note that selected error categories were non-mutually exclusive because prioritization, reasoning, and interpretation failures could co-occur in the same incorrect response. These revisions appear at lines 260-270 and lines 452-459.

## Minor Issues

**Reviewer 3 Minor Issue 1.** *Reduce speculative language in results.*

**Response.** We revised language in the Results and Discussion to avoid speculation beyond the data. Specifically, we removed the previous implication that the regional performance gap was uniformly increasing, softened statements about fundamental architectural limitations, and avoided suggesting a definitive performance ceiling for current models. The revised manuscript now describes the application gap as reproducible in this dataset but does not attribute it to a single cause. These edits appear at lines 332-343, lines 398-406, lines 539-546, and lines 617-628.

**Reviewer 3 Minor Issue 2.** *Improve figure/table clarity.*

**Response.** We improved clarity by distinguishing the two gap definitions that were previously conflated. Table 1 now identifies the mean category-level gap, while Figure 2 is described as reporting the top-model gap. The Table 1 note, Comparative Analysis subsection, and Figure 2 legend were harmonized so readers can see exactly which gap is being reported in each place. We also revised the Table 3 note to explain the interpretation of non-mutually exclusive error categories. These changes appear at lines 327-343, lines 452-459, and in the revised Figure 2 legend.

**Reviewer 3 Minor Issue 3.** *Add ethical considerations regarding clinical AI use.*

**Response.** We added a Clinical AI Ethics and Governance Considerations paragraph to the Methods. The new paragraph clarifies that the study did not involve clinical deployment, that model outputs were analyzed only as non-clinical research data, and that any future educational or clinical use of AI systems should remain subject to patient safety requirements, professional accountability, transparency, bias mitigation, human oversight, and institutional governance. This new paragraph appears at lines 288-296.

## Additional Suggestion: Related Work Updates

**Reviewer 3 Additional Suggestion.** *To strengthen the literature review with recent advancements, please consider discussing and adding the following highly relevant recent papers to your reference list:

https://arxiv.org/pdf/2605.00224

https://arxiv.org/pdf/2409.14563

https://openreview.net/pdf?id=EehtvgNXAl

https://arxiv.org/pdf/2510.12178*

**Response.** We thank the reviewer for these suggestions. In accordance with the journal’s guidance, we reviewed the suggested works for relevance rather than adding them automatically. Because several of the suggested items are broad, preprint-stage, or not directly focused on nursing licensure assessment, the Chinese NNLE, or nursing-specific clinical AI governance, we retained a focused reference list centered on the literature most directly connected to the manuscript’s aims. At the same time, we revised the future-research and methods language to reflect the reviewer’s broader point that LLM evaluation is advancing rapidly and that future work should address uncertainty-aware evaluation, alignment, instruction tuning, and parameter-efficient adaptation. The future-research language appears at lines 597-606.

We are grateful for the opportunity to revise the manuscript and believe that the revised submission is clearer, more internally consistent, and more transparent in its methodological and interpretive claims.

Sincerely,

Jionghuang Chen, MD

Corresponding Author

Department of General Surgery, Sir Run Run Shaw Hospital, Zhejiang University School of Medicine, Hangzhou, China
