# Supplementary material for: From knowledge to judgment: A three-year longitudinal analysis of artificial intelligence large language model performance on the Chinese national nurse licensing examination
Source: PLoS One. 2026 Jul 2;21(7):e0353059. doi: 10.1371/journal.pone.0353059 (PMC13327122; doi:10.1371/journal.pone.0353059)
Supplement: Peer review history

# Author comment: Author response to Decision Letter 0

**DOI:** 10.1371/journal.pone.0353059.r001

30 Aug 2025

Attachment Submitted filename: PLOSOne_Human_Subjects_Research_Checklist.docx

# Peer review: Decision Letter 0

**DOI:** 10.1371/journal.pone.0353059.r002

**Authors:** Krit Pongpirul

25 Nov 2025

PONE-D-25-46495From Knowledge to Judgment: Tracking LLM Progress on the

Chinese National Nurse Licensing Examination Over Three Years.PLOS ONE

Dear Dr. Chen,

Thank you for submitting your manuscript to PLOS ONE. After careful consideration, we feel that it has merit but does not fully meet PLOS ONE’s publication criteria as it currently stands. Therefore, we invite you to submit a revised version of the manuscript that addresses the points raised during the review process.

Please submit your revised manuscript by Jan 09 2026 11:59PM. If you will need more time than this to complete your revisions, please reply to this message or contact the journal office at plosone@plos.org. When you're ready to submit your revision, log on to https://www.editorialmanager.com/pone/ and select the 'Submissions Needing Revision' folder to locate your manuscript file.

Please include the following items when submitting your revised manuscript:

- A rebuttal letter that responds to each point raised by the academic editor and reviewer(s). You should upload this letter as a separate file labeled 'Response to Reviewers'.
- A marked-up copy of your manuscript that highlights changes made to the original version. You should upload this as a separate file labeled 'Revised Manuscript with Track Changes'.
- An unmarked version of your revised paper without tracked changes. You should upload this as a separate file labeled 'Manuscript'.

If you would like to make changes to your financial disclosure, please include your updated statement in your cover letter. Guidelines for resubmitting your figure files are available below the reviewer comments at the end of this letter.

If applicable, we recommend that you deposit your laboratory protocols in protocols.io to enhance the reproducibility of your results. Protocols.io assigns your protocol its own identifier (DOI) so that it can be cited independently in the future. For instructions see: https://journals.plos.org/plosone/s/submission-guidelines#loc-laboratory-protocols. Additionally, PLOS ONE offers an option for publishing peer-reviewed Lab Protocol articles, which describe protocols hosted on protocols.io. Read more information on sharing protocols at https://plos.org/protocols?utm_medium=editorial-email&utm_source=authorletters&utm_campaign=protocols.

We look forward to receiving your revised manuscript.

Kind regards,

Krit Pongpirul, MD, MPH, PhD.

Academic Editor

PLOS ONE

Journal requirements:

When submitting your revision, we need you to address these additional requirements.

1. Please ensure that your manuscript meets PLOS ONE's style requirements, including those for file naming. The PLOS ONE style templates can be found at

https://journals.plos.org/plosone/s/file?id=wjVg/PLOSOne_formatting_sample_main_body.pdf and

https://journals.plos.org/plosone/s/file?id=ba62/PLOSOne_formatting_sample_title_authors_affiliations.pdf

2. Please note that PLOS One has specific guidelines on code sharing for submissions in which author-generated code underpins the findings in the manuscript. In these cases, we expect all author-generated code to be made available without restrictions upon publication of the work. Please review our guidelines at https://journals.plos.org/plosone/s/materials-and-software-sharing#loc-sharing-code and ensure that your code is shared in a way that follows best practice and facilitates reproducibility and reuse.

3. Please include your tables as part of your main manuscript and remove the individual files. Please note that supplementary tables (should remain/ be uploaded) as separate "supporting information" files.

4. Thank you for stating the following financial disclosure:

[This work was supported by the Zhejiang Provincial Natural Science Foundation of China (Grant No. LQ24H160021) and Zhejiang medical and health project (Grant No. 2023KY781).].

Please state what role the funders took in the study.  If the funders had no role, please state: "The funders had no role in study design, data collection and analysis, decision to publish, or preparation of the manuscript."

If this statement is not correct you must amend it as needed.

Please include this amended Role of Funder statement in your cover letter; we will change the online submission form on your behalf.

5. In the online submission form, you indicated that [Available from the corresponding author upon reasonable request.].

All PLOS journals now require all data underlying the findings described in their manuscript to be freely available to other researchers, either 1. In a public repository, 2. Within the manuscript itself, or 3. Uploaded as supplementary information.

This policy applies to all data except where public deposition would breach compliance with the protocol approved by your research ethics board. If your data cannot be made publicly available for ethical or legal reasons (e.g., public availability would compromise patient privacy), please explain your reasons on resubmission and your exemption request will be escalated for approval.

6. PLOS requires an ORCID iD for the corresponding author in Editorial Manager on papers submitted after December 6th, 2016. Please ensure that you have an ORCID iD and that it is validated in Editorial Manager. To do this, go to ‘Update my Information’ (in the upper left-hand corner of the main menu), and click on the Fetch/Validate link next to the ORCID field. This will take you to the ORCID site and allow you to create a new iD or authenticate a pre-existing iD in Editorial Manager.

7. Please include a copy of Table 1-3 which you refer to in your text on page 22.

8. If the reviewer comments include a recommendation to cite specific previously published works, please review and evaluate these publications to determine whether they are relevant and should be cited. There is no requirement to cite these works unless the editor has indicated otherwise.

Additional Editor Comments:

Please carefully address the responses from both reviewers, particularly the concerns regarding authorship and funding.

[Note: HTML markup is below. Please do not edit.]

Reviewers' comments:

Reviewer's Responses to Questions

**Comments to the Author**

1. Is the manuscript technically sound, and do the data support the conclusions?

The manuscript must describe a technically sound piece of scientific research with data that supports the conclusions. Experiments must have been conducted rigorously, with appropriate controls, replication, and sample sizes. The conclusions must be drawn appropriately based on the data presented.

Reviewer #1: Yes

Reviewer #2: Partly

**********

2. Has the statistical analysis been performed appropriately and rigorously?

Reviewer #1: Yes

Reviewer #2: Yes

**********

3. Have the authors made all data underlying the findings in their manuscript fully available?

The PLOS Data policy requires authors to make all data underlying the findings described in their manuscript fully available without restriction, with rare exception (please refer to the Data Availability Statement in the manuscript PDF file). The data should be provided as part of the manuscript or its supporting information, or deposited to a public repository. For example, in addition to summary statistics, the data points behind means, medians and variance measures should be available. If there are restrictions on publicly sharing data—e.g. participant privacy or use of data from a third party—those must be specified.

Reviewer #1: Yes

Reviewer #2: Yes

**********

4. Is the manuscript presented in an intelligible fashion and written in standard English?

PLOS ONE does not copyedit accepted manuscripts, so the language in submitted articles must be clear, correct, and unambiguous. Any typographical or grammatical errors should be corrected at revision, so please note any specific errors here.

Reviewer #1: Yes

Reviewer #2: Yes

**********

5. Review Comments to the Author

Please use the space provided to explain your answers to the questions above. You may also include additional comments for the author, including concerns about dual publication, research ethics, or publication ethics. (Please upload your review as an attachment if it exceeds 20,000 characters)

Reviewer #1: Funding Statement Contradiction

The manuscript declares in the “Funding” section (line 504) that “This research received no specific grant funding,” yet the submission form explicitly states it was supported by two Chinese grants (Zhejiang Provincial Natural Science Foundation No. LQ24H160021 and Zhejiang Medical and Health Project No. 2023KY781). This discrepancy must be resolved—either the funding statement is inaccurate, or the submission form was misreported.

Inadequate Data Availability Compliance

Although the authors affirm in the submission form that “all data are fully available without restriction,” the manuscript’s Data Availability statement reads: “Available from the corresponding author upon reasonable request.” This phrasing violates PLOS ONE’s data policy, which requires public, immediate access (e.g., via a repository with DOI) unless a rare legal/ethical exception applies—which is not claimed here.

Authorship and Contributor Mismatch

The “Authors’ Contributions” section lists individuals with initials “T-TY” and “X-LH” (lines 507–508), but these do not correspond to any of the four named authors (Xinju Zhan, Weihua Yu, Jianshu Cai, Jionghuang Chen). This raises concerns about ghost authorship or a copy-paste error that must be corrected for transparency and accountability.

Ethics Statement Inconsistency

The online submission form marks the Ethics Statement as “N/A,” but the manuscript describes IRB exemption (#2024-NURS-089) from Sir Run Run Shaw Hospital. Since the study used publicly available exam questions and no human subjects, an exemption is appropriate—but the submission form must reflect this, not “N/A.”

Unsubstantiated Passing Threshold Claim

The authors equate 60% raw accuracy with passing the Chinese National Nurse Licensing Examination (which uses a 300-point scaled score). However, they provide no evidence linking raw percentage correct to the official scaled passing standard. Without validation from the National Medical Examination Center (NMEC), the claim that models “pass” the exam is potentially misleading.

Ambiguous Temporal Evaluation Protocol

While the study claims “temporal fidelity” (testing models only on exams released after their launch), it lacks specifics: Which exact model versions were used? Were mid-year updates (e.g., GPT-4 vs. GPT-4o) accounted for? Without a clear timeline mapping models to exam dates, the longitudinal validity is weakened.

Overstatement of Clinical Competence

Phrases like “sufficient codified nursing knowledge to pass professional licensing examinations” may imply clinical readiness. Yet the study itself shows that 43% of errors involve clinical reasoning failures and 27% involve prioritization mistakes—critical gaps in real-world care. The conclusion should more clearly emphasize that exam performance ≠ safe clinical judgment.

Missing Inference Protocol Details

The evaluation used “standardized zero-shot prompting,” but key parameters (e.g., temperature, top-p, max tokens, API vs. local inference) are not reported. These choices significantly affect LLM outputs, especially for open models like Llama 3, and must be disclosed for reproducibility.

Unclear Copyright and Redistribution Status of Question Corpus

While questions were sourced from public platforms (e.g., 21wecan.com), it is unclear whether the compiled 7,200-question dataset can be legally shared. If derived from copyrighted prep materials, public redistribution may not be permissible—requiring either repository deposition with proper licensing or clarification of public-domain status.

Reviewer #2: This report provides a comprehensive review of the manuscript titled “From Knowledge to Judgment: Tracking LLM Progress on the Chinese National Nurse Licensing Examination Over Three Years”. The study addresses a relevant and timely topic with practical implications. However, the introduction section requires a thorough clarification of theoretical framework of the study. Also, the AI systems are mentioned in many parts of the research but not prominent in the title, this raises some concerns to me, refining of the title or the writing may be required. Regarding the introduction section, it lacks a clear theoretical framework. Also, the lines 97-98, more clarification or definition is needed for the examples of Chinese- native LLMs. Concerning the methodology section, line 144-146, clarify if these domains are the only official domain studied in the Chinese nursing institutions, if not, why other domains as nursing administration or critical care nursing are not included in the exam and the study. Line 166, kindly, give example to clearly define the concept of zero-shot prompting protocols. In regard to the results section, it was concise, clear and to the point. Concerning the discussion section, in my opinion, it will be better to add a researchers’ point of view regarding the nursing licensing examinations internationally at the end, of course. Line 457-461, I think it would be good to transfer these lines to the conclusion and practical recommendation sections. Regarding the conclusion section, it is concise and to the point. However, it needs clarification for the nursing profession and for the nursing practice.

**********

6. PLOS authors have the option to publish the peer review history of their article (what does this mean?). If published, this will include your full peer review and any attached files.

If you choose “no”, your identity will remain anonymous but your review may still be made public.

**Do you want your identity to be public for this peer review?**  For information about this choice, including consent withdrawal, please see our Privacy Policy.

Reviewer #1: **Yes:** Dr. Mohammad Abuadas

Reviewer #2: **Yes:** Dr. Lareen Magdi El-Sayed Abo-Seif

**********

[NOTE: If reviewer comments were submitted as an attachment file, they will be attached to this email and accessible via the submission site. Please log into your account, locate the manuscript record, and check for the action link "View Attachments". If this link does not appear, there are no attachment files.]

To ensure your figures meet our technical requirements, please review our figure guidelines: https://journals.plos.org/plosone/s/figures

You may also use PLOS’s free figure tool, NAAS, to help you prepare publication quality figures: https://journals.plos.org/plosone/s/figures#loc-tools-for-figure-preparation.

NAAS will assess whether your figures meet our technical requirements by comparing each figure against our figure specifications.

# Author comment: Author response to Decision Letter 1

**DOI:** 10.1371/journal.pone.0353059.r003

15 Dec 2025

Response to Reviewers

Manuscript ID: PONE-D-25-46495

Title: From Knowledge to Judgment: Tracking LLM Progress on the Chinese National Nurse Licensing Examination Over Three Years

Dr. Krit Pongpirul, MD, MPH, PhD.

Academic Editor PLOS ONE

Dear Dr. Pongpirul,

We wish to express our sincere gratitude to you and the distinguished reviewers for the thorough and constructive evaluation of our manuscript. The insightful comments have been invaluable in strengthening the scientific rigor and clarity of our work. We have carefully considered each point raised and have undertaken substantial revisions to address all concerns comprehensively.

Below, we provide detailed responses to each comment from both reviewers, along with descriptions of the corresponding modifications made to the manuscript. All changes have been tracked in the revised manuscript for ease of identification.

________________________________________

RESPONSE TO JOURNAL REQUIREMENTS

1. Please ensure that your manuscript meets PLOS ONE's style requirements, including those for file naming. The PLOS ONE style templates can be found at

https://journals.plos.org/plosone/s/file?id=wjVg/PLOSOne_formatting_sample_main_body.pdf and

https://journals.plos.org/plosone/s/file?id=ba62/PLOSOne_formatting_sample_title_authors_affiliations.pdf

Response: We have carefully reviewed the PLOS ONE style templates and reformatted the manuscript to ensure full compliance with the journal's formatting guidelines, including file naming conventions, heading structures, and reference formatting. Additionally, all references throughout the manuscript have been updated and arranged according to PLOS ONE's reference style requirements.

2. Please note that PLOS One has specific guidelines on code sharing for submissions in which author-generated code underpins the findings in the manuscript. In these cases, we expect all author-generated code to be made available without restrictions upon publication of the work. Please review our guidelines at https://journals.plos.org/plosone/s/materials-and-software-sharing#loc-sharing-code and ensure that your code is shared in a way that follows best practice and facilitates reproducibility and reuse.

Response: We acknowledge the journal's guidelines on code sharing. As our study involved API-based evaluation of commercial and open-source language models rather than custom-developed code, we have clarified in the revised manuscript that no proprietary code was developed for this research. The standardized prompting protocols and evaluation procedures have been fully described in the Methods section to ensure complete reproducibility.

3. Please include your tables as part of your main manuscript and remove the individual files. Please note that supplementary tables (should remain/ be uploaded) as separate "supporting information" files.

Response: We have integrated Tables 1-3 directly into the main manuscript body at appropriate locations following their first textual reference, as requested. The tables have been removed as separate files.

4. Thank you for stating the following financial disclosure:

[This work was supported by the Zhejiang Provincial Natural Science Foundation of China (Grant No. LQ24H160021) and Zhejiang medical and health project (Grant No. 2023KY781).].

Please state what role the funders took in the study. If the funders had no role, please state: "The funders had no role in study design, data collection and analysis, decision to publish, or preparation of the manuscript."

If this statement is not correct you must amend it as needed.

Please include this amended Role of Funder statement in your cover letter; we will change the online submission form on your behalf.

Response: We sincerely apologize for this discrepancy. The funding statement in the original manuscript was erroneous. This work was indeed supported by the Zhejiang Provincial Natural Science Foundation of China (Grant No. LQ24H160021) and Zhejiang Medical and Health Project (Grant No. 2023KY781). We have corrected the Funding section in the revised manuscript to accurately reflect this support. Regarding the role of funders: "The funders had no role in study design, data collection and analysis, decision to publish, or preparation of the manuscript."

5. In the online submission form, you indicated that [Available from the corresponding author upon reasonable request.].

All PLOS journals now require all data underlying the findings described in their manuscript to be freely available to other researchers, either 1. In a public repository, 2. Within the manuscript itself, or 3. Uploaded as supplementary information.

This policy applies to all data except where public deposition would breach compliance with the protocol approved by your research ethics board. If your data cannot be made publicly available for ethical or legal reasons (e.g., public availability would compromise patient privacy), please explain your reasons on resubmission and your exemption request will be escalated for approval.

Response: We acknowledge that our previous data availability statement required clarification regarding PLOS ONE's data policy. The data underlying this study are subject to institutional data governance policies at Sir Run Run Shaw Hospital, Zhejiang University School of Medicine, which restrict public dissemination of research datasets without prior institutional approval. This policy exists to ensure appropriate oversight of data sharing and compliance with institutional research governance standards.

Therefore, we respectfully request an exemption from the standard data availability requirements based on institutional policy restrictions. To ensure transparency and facilitate reproducibility within these constraints, we have revised our Data Availability Statement to read: "The data underlying this study are subject to institutional data governance policies at Sir Run Run Shaw Hospital, Zhejiang University School of Medicine. Data requests may be sent to the institutional data contact: Ms. Jiang Miaomiao (jiang0304@zju.edu.cn), Nursing Department, Sir Run Run Shaw Hospital, Zhejiang University School of Medicine. Qualified researchers may access data subject to institutional approval and completion of a data sharing agreement. Researchers may also contact the corresponding author, Dr. Jionghuang Chen (zxj6682@126.com), for coordination of data access requests. The institutional repository ensures long-term data preservation per the hospital's 10-year research data retention policy."

This approach balances PLOS ONE's commitment to transparency with our institutional obligations.

6. PLOS requires an ORCID iD for the corresponding author in Editorial Manager on papers submitted after December 6th, 2016. Please ensure that you have an ORCID iD and that it is validated in Editorial Manager. To do this, go to ‘Update my Information’ (in the upper left-hand corner of the main menu), and click on the Fetch/Validate link next to the ORCID field. This will take you to the ORCID site and allow you to create a new iD or authenticate a pre-existing iD in Editorial Manager.

Response: The corresponding author has validated the ORCID iD: 0009-0007-2618-6500 in Editorial Manager as requested.

7. Please include a copy of Table 1-3 which you refer to in your text on page 22.

Response: Tables 1-3 are now included within the main manuscript text at the locations where they are first referenced.

8. If the reviewer comments include a recommendation to cite specific previously published works, please review and evaluate these publications to determine whether they are relevant and should be cited. There is no requirement to cite these works unless the editor has indicated otherwise.

Response: We have carefully evaluated all citations recommended by the reviewers and incorporated those deemed relevant and scientifically appropriate into the revised manuscript.

________________________________________

RESPONSE TO REVIEWER #1

We are deeply appreciative of Reviewer #1's meticulous examination of our manuscript. The reviewer has identified several critical issues that required immediate attention, and we have addressed each concern with the utmost seriousness.

Comment 1: Funding Statement Contradiction

The manuscript declares in the "Funding" section (line 504) that "This research received no specific grant funding," yet the submission form explicitly states it was supported by two Chinese grants.

Response: We sincerely apologize for this significant oversight, which resulted from an administrative error during manuscript preparation. The correct information is that this research was supported by the Zhejiang Provincial Natural Science Foundation of China (Grant No. LQ24H160021) and Zhejiang Medical and Health Project (Grant No. 2023KY781). We have corrected the Funding section to read: "This work was supported by the Zhejiang Provincial Natural Science Foundation of China (Grant No. LQ24H160021) and the Zhejiang Medical and Health Project (Grant No. 2023KY781). The funders had no role in study design, data collection and analysis, decision to publish, or preparation of the manuscript."

Comment 2: Inadequate Data Availability Compliance

The manuscript's Data Availability statement reads: "Available from the corresponding author upon reasonable request." This phrasing violates PLOS ONE's data policy.

Response: We appreciate the reviewer highlighting this important issue and wish to provide clarification. The data underlying this study are subject to institutional data governance policies at Sir Run Run Shaw Hospital, Zhejiang University School of Medicine, which restrict public dissemination of research datasets without prior institutional approval. This institutional policy exists to ensure appropriate oversight of data sharing and compliance with research governance standards.

We have revised the Data Availability Statement in the Declarations section to transparently explain this restriction: "The data underlying this study are subject to institutional data governance policies at Sir Run Run Shaw Hospital, Zhejiang University School of Medicine. Data requests may be sent to the institutional data contact: Ms. Jiang Miaomiao (jiang0304@zju.edu.cn), Nursing Department, Sir Run Run Shaw Hospital, Zhejiang University School of Medicine. Qualified researchers may access data subject to institutional approval and completion of a data sharing agreement. Researchers may also contact the corresponding author, Dr. Jionghuang Chen (zxj6682@126.com), for coordination of data access requests. The institutional repository ensures long-term data preservation per the hospital's 10-year research data retention policy.""

This revised statement complies with PLOS ONE's policy for cases where institutional restrictions prevent full public data sharing, as it clearly specifies the nature of the restriction and provides a clear pathway for qualified researchers to request data access through appropriate channels.

Comment 3: Authorship and Contributor Mismatch

The "Authors' Contributions" section lists individuals with initials "T-TY" and "X-LH" (lines 507-508), but these do not correspond to any of the four named authors.

Response: We are grateful for identifying this serious error, which occurred due to a copy-paste mistake during manuscript compilation from an earlier draft. We have thoroughly revised the Authors' Contributions section to accurately reflect the contributions of the four named authors: "X-JZ and J-HC contributed equally to study design, data collection, and manuscript preparation. J-SC performed statistical analyses and figure preparation. W-HY supervised the study and provided critical manuscript revisions. All authors read and approved the final manuscript."

Comment 4: Ethics Statement Inconsistency

The online submission form marks the Ethics Statement as "N/A," but the manuscript describes IRB exemption (#2024-NURS-089).

Response: We acknowledge this inconsistency and have rectified the submission form to accurately reflect our IRB exemption status. The study appropriately received exemption from the Institutional Research Ethics Committee at Sir Run Run Shaw Hospital (Protocol #2024-NURS-089) as it involved only publicly available educational materials and computational model evaluation with no human subjects. We have updated the online submission form accordingly.

Comment 5: Unsubstantiated Passing Threshold Claim

The authors equate 60% raw accuracy with passing the Chinese National Nurse Licensing Examination (which uses a 300-point scaled score). However, they provide no evidence linking raw percentage correct to the official scaled passing standard.

Response: We appreciate this important methodological concern. We have substantially revised our discussion of the passing threshold to address this limitation transparently. The revised manuscript now includes the following clarification in the Methods section: "It should be noted that the NNLE employs a scaled scoring system with a passing threshold of 300 points. While the precise algorithm for converting raw scores to scaled scores is not publicly disclosed by the National Medical Examination Center, historical analysis of examination outcomes and preparatory materials consistently indicates that approximately 60% raw accuracy corresponds to passing performance. We acknowledge this as a methodological limitation and have adopted this threshold as an approximate benchmark rather than an exact equivalence." Additionally, we have moderated our claims regarding "passing" performance throughout the manuscript to reflect this uncertainty.

Comment 6: Ambiguous Temporal Evaluation Protocol

While the study claims "temporal fidelity," it lacks specifics: Which exact model versions were used? Were mid-year updates accounted for?

Response: We thank the reviewer for highlighting this important gap in methodological transparency. We have added comprehensive supplementary materials detailing the exact model versions, API endpoints, and access dates for each evaluation. Supplementary Table S1 provides complete model version documentation for all international and Chinese-native LLMs, including specific API endpoints, parameter counts, release dates, evaluation periods, and training data cutoffs. Supplementary Table S2 documents the standardized inference parameters, and Supplementary Table S3 details the hardware and software specifications for local inference of open-source models. Furthermore, we have expanded the Methods section to include: "To ensure temporal fidelity, we documented specific model versions and API endpoints for each evaluation period. For models with mid-year updates (e.g., GPT-4 to GPT-4-turbo), we utilized the version available at the time of each examination cycle's evaluation. Specifically, GPT-4 evaluations in 2023 employed the gpt-4-0314 endpoint, while 2024 evaluations utilized gpt-4-0613. Complete version documentation is provided in Supplementary Table S1-S3."

Comment 7: Overstatement of Clinical Competence

Phrases like "sufficient codified nursing knowledge to pass professional licensing examinations" may imply clinical readiness. The conclusion should more clearly emphasize that exam performance ≠ safe clinical judgment.

Response: We concur entirely with this concern and have substantially revised our language throughout the manuscript to avoid any implication of clinical readiness. The revised Conclusion now emphasizes: "Critically, the ability to pass a professional licensing examination should not be interpreted as evidence of clinical competence or readiness for autonomous patient care. Our error analysis reveals that 43% of mistakes among top-performing models involved clinical reasoning failures and 27% involved prioritization errors—precisely the capabilities most essential for safe patient care. These findings underscore that current LLMs function as sophisticated knowledge repositories rather than clinical reasoning agents, and any deployment in healthcare settings must incorporate robust human oversight frameworks."

Comment 8: Missing Inference Protocol Details

The evaluation used "standardized zero-shot prompting," but key parameters (e.g., temperature, top-p, ma

Attachment Submitted filename: Response Letter.docx

# Peer review: Decision Letter 1

**DOI:** 10.1371/journal.pone.0353059.r004

**Authors:** Krit Pongpirul

20 May 2026

PONE-D-25-46495R1

From Knowledge to Judgment: A Three-Year Longitudinal Analysis of Artificial Intelligence Large Language Model Performance on the Chinese National Nurse Licensing Examination

PLOS One

Dear Dr. Chen,

Thank you for submitting your manuscript to PLOS ONE. I appreciate the depth of your engagement with reviewer feedback. After careful consideration, we feel that it has merit but does not fully meet PLOS ONE’s publication criteria as it currently stands. Therefore, we invite you to submit a revised version of the manuscript that addresses the points below:

1. **Reconcile contradictory performance-gap figures.**  The abstract states that the Chinese-native versus international performance gap increased from 1.6% (2023) to 3.8% (2025), and the Discussion repeats this. Table 1 reports performance gaps of 6.1% (2023), 4.2% (2024), and 3.0% (2025) — a decreasing gap. The Results body separately notes a peak of 4.5 percentage points in 2023. Please clarify which figures are correct and revise the abstract, results narrative, discussion, and cover letter so that they tell a single consistent story. If the direction of the gap is genuinely changing over time, the abstract claim of an "increasing" gap is contradicted by Table 1.
2. **Correct the section-level accuracy figures in the abstract.**  The abstract reports Professional Practice = 83.5% and Practical Skills = 74.6%. The Results body and Table 2 indicate Professional Practice = 81.6% and Practical Skills = 70.9%; the 83.5% figure corresponds to the Ethics & Regulations domain rather than the Professional Practice section. Please correct the abstract to use the section-level figures consistent with the Results.
3. **Confirm Supplementary Tables S1–S3 are uploaded.**  Your response to Reviewer #1 Comments 6 and 8 indicates that Supplementary Tables S1 (model versions, API endpoints, training data cutoffs), S2 (inference parameters), and S3 (hardware/software specifications) have been added. These are referenced in the Methods but do not appear in the merged submission file I reviewed. Please confirm they are attached as Supporting Information.
4. **Reconcile the corpus arithmetic with the study span.**  The Methods state that the corpus spans July 2022 to June 2025 and contains 7,200 questions, with a breakdown of 2,200 (2022), 2,400 (2023), and 2,600 (2024). The 2025 examination cycle is missing from this breakdown despite featuring centrally in Table 1 and throughout the Results. Please either add the 2025 question count or clarify the corpus composition.

Please submit your revised manuscript by Jul 04 2026 11:59PM. If you will need more time than this to complete your revisions, please reply to this message or contact the journal office at plosone@plos.org. When you're ready to submit your revision, log on to https://www.editorialmanager.com/pone/ and select the 'Submissions Needing Revision' folder to locate your manuscript file.

Please include the following items when submitting your revised manuscript:

- A letter that responds to each point raised by the academic editor and reviewer(s). You should upload this letter as a separate file labeled 'Response to Reviewers'.
- A marked-up copy of your manuscript that highlights changes made to the original version. You should upload this as a separate file labeled 'Revised Manuscript with Track Changes'.
- An unmarked version of your revised paper without tracked changes. You should upload this as a separate file labeled 'Manuscript'.

If you would like to make changes to your financial disclosure, please include your updated statement in your cover letter. Guidelines for resubmitting your figure files are available below the reviewer comments at the end of this letter.

If applicable, we recommend that you deposit your laboratory protocols in protocols.io to enhance the reproducibility of your results. Protocols.io assigns your protocol its own identifier (DOI) so that it can be cited independently in the future. For instructions see: https://journals.plos.org/plosone/s/submission-guidelines#loc-laboratory-protocols. Additionally, PLOS ONE offers an option for publishing peer-reviewed Lab Protocol articles, which describe protocols hosted on protocols.io. Read more information on sharing protocols at https://plos.org/protocols?utm_medium=editorial-email&utm_source=authorletters&utm_campaign=protocols.

As the corresponding author, your ORCID iD is verified in the submission system and will appear in the published article. PLOS supports the use of ORCID, and we encourage all coauthors to register for an ORCID iD and use it as well. Please encourage your coauthors to verify their ORCID iD within the submission system before final acceptance, as unverified ORCID iDs will not appear in the published article. *Only*  the individual author can complete the verification step; PLOS staff *cannot*  verify ORCID iDs on behalf of authors.

We look forward to receiving your revised manuscript.

Kind regards,

Isaac Amankwaa, Ph.D.

Academic Editor

PLOS One

**Journal Requirements:**

1. If the reviewer comments include a recommendation to cite specific previously published works, please review and evaluate these publications to determine whether they are relevant and should be cited. There is no requirement to cite these works unless the editor has indicated otherwise.

2. Please review your reference list to ensure that it is complete and correct. If you have cited papers that have been retracted, please include the rationale for doing so in the manuscript text, or remove these references and replace them with relevant current references. Any changes to the reference list should be mentioned in the rebuttal letter that accompanies your revised manuscript. If you need to cite a retracted article, indicate the article’s retracted status in the References list and also include a citation and full reference for the retraction notice.

[Note: HTML markup is below. Please do not edit.]

Reviewers' comments:

Reviewer's Responses to Questions

**Comments to the Author**

1. If the authors have adequately addressed your comments raised in a previous round of review and you feel that this manuscript is now acceptable for publication, you may indicate that here to bypass the “Comments to the Author” section, enter your conflict of interest statement in the “Confidential to Editor” section, and submit your "Accept" recommendation.

Reviewer #2: All comments have been addressed

Reviewer #3: (No Response)

**********

2. Is the manuscript technically sound, and do the data support the conclusions?

The manuscript must describe a technically sound piece of scientific research with data that supports the conclusions. Experiments must have been conducted rigorously, with appropriate controls, replication, and sample sizes. The conclusions must be drawn appropriately based on the data presented.

Reviewer #2: Yes

Reviewer #3: Yes

**********

3. Has the statistical analysis been performed appropriately and rigorously?

Reviewer #2: Yes

Reviewer #3: (No Response)

**********

4. Have the authors made all data underlying the findings in their manuscript fully available?

The PLOS Data policy requires authors to make all data underlying the findings described in their manuscript fully available without restriction, with rare exception (please refer to the Data Availability Statement in the manuscript PDF file). The data should be provided as part of the manuscript or its supporting information, or deposited to a public repository. For example, in addition to summary statistics, the data points behind means, medians and variance measures should be available. If there are restrictions on publicly sharing data—e.g. participant privacy or use of data from a third party—those must be specified.

Reviewer #2: Yes

Reviewer #3: Yes

**********

5. Is the manuscript presented in an intelligible fashion and written in standard English?

PLOS ONE does not copyedit accepted manuscripts, so the language in submitted articles must be clear, correct, and unambiguous. Any typographical or grammatical errors should be corrected at revision, so please note any specific errors here.

Reviewer #2: Yes

Reviewer #3: Yes

**********

6. Review Comments to the Author

Please use the space provided to explain your answers to the questions above. You may also include additional comments for the author, including concerns about dual publication, research ethics, or publication ethics. (Please upload your review as an attachment if it exceeds 20,000 characters)

Reviewer #2: (No Response)

Reviewer #3: Major Issues

Methods insufficiently described — Dataset source, prompting strategy, and reproducibility details are unclear.

Possible data leakage not adequately addressed.

Overinterpretation of results — Exam performance should not be equated with clinical competence.

Statistical methods need clarification (assumptions, multiple comparisons).

Error analysis is strong but requires explanation of coding procedures and reviewer reliability.

Minor Issues

Reduce speculative language in results.

Improve figure/table clarity.

Add ethical considerations regarding clinical AI use.

Additional Suggestion: Related Work Updates

To strengthen the literature review with recent advancements, please consider discussing and adding the following highly relevant recent papers to your reference list:

https://arxiv.org/pdf/2605.00224

https://arxiv.org/pdf/2409.14563

https://openreview.net/pdf?id=EehtvgNXAl

https://arxiv.org/pdf/2510.12178

**********

7. PLOS authors have the option to publish the peer review history of their article (what does this mean?). If published, this will include your full peer review and any attached files.

If you choose “no”, your identity will remain anonymous but your review may still be made public.

**Do you want your identity to be public for this peer review?**  For information about this choice, including consent withdrawal, please see our Privacy Policy.

Reviewer #2: **Yes:** Dr. Lareen Magdi El-Sayed Abo-Seif

Reviewer #3: No

**********

[NOTE: If reviewer comments were submitted as an attachment file, they will be attached to this email and accessible via the submission site. Please log into your account, locate the manuscript record, and check for the action link "View Attachments". If this link does not appear, there are no attachment files.]

To ensure your figures meet our technical requirements, please review our figure guidelines: https://journals.plos.org/plosone/s/figures

You may also use PLOS’s free figure tool, NAAS, to help you prepare publication quality figures: https://journals.plos.org/plosone/s/figures#loc-tools-for-figure-preparation.

NAAS will assess whether your figures meet our technical requirements by comparing each figure against our figure specifications.

# Author comment: Author response to Decision Letter 2

**DOI:** 10.1371/journal.pone.0353059.r005

22 May 2026

Response to the Academic Editor and Reviewers

Dear Dr. Amankwaa,

On behalf of all authors, I sincerely thank you and the reviewers for the careful re-evaluation of our manuscript and for the constructive guidance provided in this revision round. We have revised the manuscript with focused attention to numerical consistency, corpus accounting, reproducibility, data leakage safeguards, statistical transparency, error-coding reliability, figure and table clarity, and the ethical interpretation of artificial intelligence performance in nursing contexts. We also confirm that Supplementary Tables S1-S3 are submitted as a separate Supporting Information file.

For transparency, the editor and reviewer comments are reproduced below using the same wording as presented in the decision letter. Where Reviewer 3 provided a consolidated list of issues, we separated the list into individual response items while preserving the reviewer’s wording. The line references below refer to the clean revised manuscript with line numbering. Because line numbering can shift after Editorial Manager regenerates the submission PDF, we also identify the relevant manuscript section, table, or figure legend for each change.

In preparing the final submission package, we rebuilt the manuscript from the original Word file so that the EndNote citation field structure remained intact. This response letter is a standalone document and does not contain EndNote field codes.

Response to the Academic Editor

Academic Editor Comment 1. Reconcile contradictory performance-gap figures. The abstract states that the Chinese-native versus international performance gap increased from 1.6% (2023) to 3.8% (2025), and the Discussion repeats this. Table 1 reports performance gaps of 6.1% (2023), 4.2% (2024), and 3.0% (2025) — a decreasing gap. The Results body separately notes a peak of 4.5 percentage points in 2023. Please clarify which figures are correct and revise the abstract, results narrative, discussion, and cover letter so that they tell a single consistent story. If the direction of the gap is genuinely changing over time, the abstract claim of an "increasing" gap is contradicted by Table 1.

Response. We appreciate this careful observation and agree that the previous revision unintentionally conflated two different gap definitions. After rechecking the calculations, we clarified that Table 1 reports the mean category-level difference between Chinese-native models and international models, with gaps of 6.1, 4.2, and 3.0 percentage points from 2023 to 2025. Figure 2 and the relevant Results narrative report the top-model gap, defined as the difference between the single highest-performing Chinese-native model and the single highest-performing international model in each year, with values of 4.5, 3.0, and 3.8 percentage points from 2023 to 2025. We therefore removed the prior statement that the gap increased over time and revised the Abstract, Results, Discussion, Table 1 note, and Figure 2 legend so that the manuscript presents one coherent interpretation: the mean category-level advantage decreased over time, while the top-model advantage remained persistent but non-monotonic. These revisions appear in the Abstract Results paragraph at lines 48-58, the Table 1 note at lines 327-330, the Comparative Analysis subsection at lines 332-343, the Discussion paragraph on regional optimization at lines 512-524, and the revised Figure 2 legend.

Academic Editor Comment 2. Correct the section-level accuracy figures in the abstract. The abstract reports Professional Practice = 83.5% and Practical Skills = 74.6%. The Results body and Table 2 indicate Professional Practice = 81.6% and Practical Skills = 70.9%; the 83.5% figure corresponds to the Ethics & Regulations domain rather than the Professional Practice section. Please correct the abstract to use the section-level figures consistent with the Results.

Response. We agree and have corrected the Abstract to match Table 2 and the Results body. The Professional Practice section is now reported as 81.6% average accuracy, and the Practical Skills section is now reported as 70.9% average accuracy. The 83.5% value is retained only for the Ethics & Regulations domain, where it belongs. This correction appears in the Abstract Results paragraph at lines 54-56 and is consistent with the Section-Specific Performance Patterns subsection at lines 393-404 and Table 2.

Academic Editor Comment 3. Confirm Supplementary Tables S1–S3 are uploaded. Your response to Reviewer #1 Comments 6 and 8 indicates that Supplementary Tables S1 (model versions, API endpoints, training data cutoffs), S2 (inference parameters), and S3 (hardware/software specifications) have been added. These are referenced in the Methods but do not appear in the merged submission file I reviewed. Please confirm they are attached as Supporting Information.

Response. We confirm that Supplementary Tables S1, S2, and S3 are included in the separate Supporting Information file submitted with this revision. To make this explicit for readers and editorial staff, we revised the Technical Specifications paragraph to state that complete version documentation, API endpoints, parameter counts, training data cutoffs, inference parameters, and hardware/software specifications are provided in Supplementary Tables S1-S3 and submitted separately as Supporting Information. This clarification appears at lines 186-196. The separate supplementary file contains Tables S1-S3 in full.

Academic Editor Comment 4. Reconcile the corpus arithmetic with the study span. The Methods state that the corpus spans July 2022 to June 2025 and contains 7,200 questions, with a breakdown of 2,200 (2022), 2,400 (2023), and 2,600 (2024). The 2025 examination cycle is missing from this breakdown despite featuring centrally in Table 1 and throughout the Results. Please either add the 2025 question count or clarify the corpus composition.

Response. We agree that the previous corpus description was incomplete. We corrected the Methods and Abstract to state that the validated corpus comprised 9,800 questions across four examination years: 2,200 from 2022, 2,400 from 2023, and 2600 questions in each of 2024 and 2025. This correction aligns the corpus description with the 2025 analyses reported in Tables 1 and 2 and throughout the Results. The Abstract Methods paragraph was revised at lines 40-47, and the Final Corpus Characteristics paragraph was revised at lines 166-179.

Response to Journal Requirements

Journal Requirement 1. If the reviewer comments include a recommendation to cite specific previously published works, please review and evaluate these publications to determine whether they are relevant and should be cited. There is no requirement to cite these works unless the editor has indicated otherwise.

Response. We reviewed and evaluated the papers suggested by Reviewer 3 under this guidance. Because the suggested works are broad or preprint-stage contributions and are not all directly focused on nursing licensure examinations, the Chinese NNLE, or clinical AI governance in nursing practice, we did not add them automatically. Instead, we retained a focused reference list centered on nursing education, medical examination performance, clinical AI evaluation, and healthcare AI governance, while revising the manuscript language to acknowledge rapidly evolving work on uncertainty-aware evaluation, alignment, instruction following, and model adaptation. This approach maintains the manuscript’s disciplinary focus while addressing the reviewer’s concern that the field is advancing quickly.

Journal Requirement 2. Please review your reference list to ensure that it is complete and correct. If you have cited papers that have been retracted, please include the rationale for doing so in the manuscript text, or remove these references and replace them with relevant current references. Any changes to the reference list should be mentioned in the rebuttal letter that accompanies your revised manuscript. If you need to cite a retracted article, indicate the article’s retracted status in the References list and also include a citation and full reference for the retraction notice.

Response. We reviewed the reference list for completeness, formatting consistency, and relevance to the revised manuscript. We did not intentionally cite any retracted work. No retracted article is required for the argument of the manuscript, and no retraction notice needed to be added. The response to Reviewer 3 below explains how we evaluated the suggested recent literature and why the final reference list remains focused on sources most directly relevant to nursing licensure, healthcare AI assessment, and clinical governance.

Response to Reviewer 2

Reviewer 2 Comment. All comments have been addressed

Response. We sincerely thank Reviewer 2 for confirming that all previous comments have been addressed and for affirming that the manuscript is technically sound, statistically appropriate, supported by available data, and written in clear English. We made no additional changes specifically in response to Reviewer 2 beyond the final editorial corrections, consistency checks, and response-letter refinements described in this document.

Response to Reviewer 3

Reviewer 3 provided a consolidated list of major issues, minor issues, and a related-work suggestion. We respond to each point individually below, preserving the wording of the reviewer’s comments while explaining the exact revisions made in the manuscript.

Major Issues

Reviewer 3 Major Issue 1. Methods insufficiently described — Dataset source, prompting strategy, and reproducibility details are unclear.

Response. We thank the reviewer for identifying areas where the Methods needed greater precision. We expanded the Methods without changing the manuscript structure. The Data Source Identification and Final Corpus Characteristics sections now give a corrected corpus count and year-level distribution. The Technical Specifications paragraph now explicitly directs readers to Supplementary Tables S1-S3 for model versions, API endpoints, parameter counts, training data cutoffs, inference parameters, and hardware/software specifications. The prompting strategy remains zero-shot, but the Evaluation Protocol section now more clearly explains the standardized Chinese prompt, response extraction, and deterministic inference settings. These changes appear at lines 166-196 and lines 203-215. Additional reproducibility details, including the temporal fidelity and data leakage safeguards, appear at lines 223-233.

Reviewer 3 Major Issue 2. Possible data leakage not adequately addressed.

Response. We agree that data leakage is a critical concern in evaluations of contemporary LLMs, particularly because the full training corpora of proprietary and open-weight models are not completely transparent. We revised the Methods to state this limitation explicitly and to explain the safeguards used in the study. These safeguards included evaluating models only under the specified temporal fidelity protocol, documenting model version strings and training data cutoffs where available, screening questions for unstable or unattributable source material, and checking for duplicate or verbatim publicly indexed items where feasible. We also clarified that residual exposure cannot be eliminated with absolute certainty and should be considered a limitation of the study. These revisions appear at lines 223-233.

Reviewer 3 Major Issue 3. Overinterpretation of results — Exam performance should not be equated with clinical competence.

Response. We fully agree with this concern and strengthened the manuscript accordingly. The Abstract, Discussion, and Conclusion now use more careful language that frames model output as written examination performance rather than clinical competence, clinical readiness, or autonomous practice capability. We also softened deterministic statements about architecture and performance ceilings. For example, the Practical Skills interpretation now states that the study cannot determine whether the observed application gap arises primarily from architecture, training data, prompting, item type, or examination structure. This revision appears at lines 398-406. The Discussion of error patterns was similarly tempered at lines 539-546. The revised Conclusion states explicitly that written examination performance should not be interpreted as evidence of clinical competence or readiness for autonomous patient care, as shown at lines 617-628.

Reviewer 3 Major Issue 4. Statistical methods need clarification (assumptions, multiple comparisons).

Response. We revised the Statistical Testing Procedures paragraph to clarify the analytical assumptions and correction procedures. The revised text now specifies residual diagnostics, Shapiro-Wilk testing for normality, Levene testing for homogeneity of variance, the use of Welch or nonparametric sensitivity analyses when assumptions were not met, and Bonferroni correction for multiple comparisons. These changes appear at lines 250-259 and strengthen the reproducibility and interpretability of the reported statistical comparisons.

Reviewer 3 Major Issue 5. Error analysis is strong but requires explanation of coding procedures and reviewer reliability.

Response. We appreciate the reviewer’s positive assessment of the error analysis and agree that the coding procedures required clearer reporting. We revised the Qualitative Analysis Framework to describe the a priori codebook, pilot coding of 100 incorrect responses, independent dual coding, consensus resolution, and reviewer reliability. Cohen’s kappa before consensus was κ = 0.86, indicating strong agreement. We also clarified in the Table 3 note that selected error categories were non-mutually exclusive because prioritization, reasoning, and interpretation failures could co-occur in the same incorrect response. These revisions appear at lines 260-270 and lines 452-459.

Minor Issues

Reviewer 3 Minor Issue 1. Reduce speculative language in results.

Response. We revised language in the Results and Discussion to avoid speculation beyond the data. Specifically, we removed the previous implication that the regional performance gap was uniformly increasing, softened statements about fundamental architectural limitations, and avoided suggesting a definitive performance ceiling for current models. The revised manuscript now describes the application gap as reproducible in this dataset but does not attribute it to a single cause. These edits appear at lines 332-343, lines 398-406, lines 539-546, and lines 617-628.

Reviewer 3 Minor Issue 2. Improve figure/table clarity.

Response. We improved clarity by distinguishing the two gap definitions that were previously conflated. Table 1 now identifies the mean category-level gap, while Figure 2 is described as reporting the top-model gap. The Table 1 note, Comparative Analysis subsection, and Figure 2 legend were harmonized so readers can see exactly which gap is being reported in each place. We also revised the Table 3 note to explain the interpretation of non-mutually exclusive error categories. These changes appear at lines 327-343, lines 452-459, and in the revised Figure 2 legend.

Reviewer 3 Minor Issue 3. Add ethical considerations regarding clinical AI use.

Response. We added a Clinical AI Ethics and Governance Considerations paragraph to the Methods. The new paragraph clarifies that the study did not involve clinical deployment, that model outputs were analyzed only as non-clinical research data, and that any future educational or clinical use of AI systems should remain subject to patient safety requirements, professional accountability, transparency, bias mitigation, human oversight, and institutional governance. This new paragraph appears at lines 288-296.

Additional Suggestion: Related Work Updates

Reviewer 3 Additional Suggestion. To strengthen the literature review with recent advancements, please consider discussing and adding the following highly relevant recent papers to your reference list:

https://arxiv.org/pdf/2605.00224

https://arxiv.org/pdf/2409.14563

https://openreview.net/pdf?id=EehtvgNXAl

https://arxiv.org/pdf/2510.12178

Response. We thank the reviewer for these suggestions. In ac

Attachment Submitted filename: Response_Letter_auresp_2.docx

# Editor report: Decision Letter 2

**DOI:** 10.1371/journal.pone.0353059.r006

**Authors:** Isaac Amankwaa

18 Jun 2026

From Knowledge to Judgment: A Three-Year Longitudinal Analysis of Artificial Intelligence Large Language Model Performance on the Chinese National Nurse Licensing Examination

PONE-D-25-46495R2

Dear Dr. Chen,

We’re pleased to inform you that your manuscript has been judged scientifically suitable for publication and will be formally accepted for publication once it meets all outstanding technical requirements.

Within one week, you’ll receive an e-mail detailing the required amendments. When these have been addressed, you’ll receive a formal acceptance letter and your manuscript will be scheduled for publication.

An invoice will be generated when your article is formally accepted. Please note, if your institution has a publishing partnership with PLOS and your article meets the relevant criteria, all or part of your publication costs will be covered. Please make sure your user information is up-to-date by logging into Editorial Manager at Editorial Manager® and clicking the ‘Update My Information' link at the top of the page. For questions related to billing, please contact billing support.

If your institution or institutions have a press office, please notify them about your upcoming paper to help maximize its impact. If they’ll be preparing press materials, please inform our press team as soon as possible -- no later than 48 hours after receiving the formal acceptance. Your manuscript will remain under strict press embargo until 2 pm Eastern Time on the date of publication. For more information, please contact onepress@plos.org.

Kind regards,

Isaac Amankwaa, Ph.D.

Academic Editor

PLOS One

Additional Editor Comments (optional):

Reviewers' comments:

# Editor report: Acceptance letter

**DOI:** 10.1371/journal.pone.0353059.r007

**Authors:** Isaac Amankwaa

PONE-D-25-46495R2

PLOS One

Dear Dr. Chen,

I'm pleased to inform you that your manuscript has been deemed suitable for publication in PLOS One. Congratulations! Your manuscript is now being handed over to our production team.

At this stage, our production department will prepare your paper for publication. This includes ensuring the following:

* All references, tables, and figures are properly cited

* All relevant supporting information is included in the manuscript submission,

* There are no issues that prevent the paper from being properly typeset

You will receive further instructions from the production team, including instructions on how to review your proof when it is ready. Please keep in mind that we are working through a large volume of accepted articles, so please give us a few days to review your paper and let you know the next and final steps.

Lastly, if your institution or institutions have a press office, please let them know about your upcoming paper now to help maximize its impact. If they'll be preparing press materials, please inform our press team within the next 48 hours. Your manuscript will remain under strict press embargo until 2 pm Eastern Time on the date of publication. For more information, please contact onepress@plos.org.

You will receive an invoice from PLOS for your publication fee after your manuscript has reached the completed accept phase. If you receive an email requesting payment before acceptance or for any other service, this may be a phishing scheme. Learn how to identify phishing emails and protect your accounts at https://explore.plos.org/phishing.

If we can help with anything else, please email us at customercare@plos.org.

Thank you for submitting your work to PLOS ONE and supporting open access.

Kind regards,

PLOS ONE Editorial Office Staff

on behalf of

Dr. Isaac Amankwaa

Academic Editor

PLOS One
